# A simple MiMIC-based approach for tagging endogenous genes to visualise live transcription in *Drosophila*

**DOI:** 10.1242/dev.204294

**Published:** 2024-12-16

**Authors:** Lauren Forbes Beadle, Catherine Sutcliffe, Hilary L. Ashe

**Affiliations:** Faculty of Biology, Medicine and Health, The University of Manchester, Manchester M13 9PT, UK

**Keywords:** Transcription, Live imaging, *Drosophila*, MS2-MCP system, PP7-PCP system, MiMIC

## Abstract

Live imaging of transcription in the *Drosophila* embryo using the MS2 or PP7 systems is transforming our understanding of transcriptional regulation. However, insertion of MS2/PP7 stem-loops into endogenous genes requires laborious CRISPR genome editing. Here, we exploit the previously described Minos-mediated integration cassette (MiMIC) transposon system in *Drosophila* to establish a method for simply and rapidly inserting MS2/PP7 cassettes into any of the thousands of genes carrying a MiMIC insertion. In addition to generating a variety of stem-loop donor fly stocks, we have made new stocks expressing the complementary coat proteins fused to different fluorescent proteins. We show the utility of this MiMIC-based approach by MS2/PP7 tagging of endogenous genes and the long non-coding RNA *roX1*, then imaging their transcription in living embryos. We also present live transcription data from larval brains, the wing disc and ovary, thereby extending the tissues that can be studied using the MS2/PP7 system. Overall, this first high-throughput method for tagging mRNAs in *Drosophila* will facilitate the study of transcription dynamics of thousands of endogenous genes in a range of *Drosophila* tissues.

## Teaser

**Summary:** A new resource that utilises MiMIC lines for endogenous gene tagging with MS2 and PP7 loops can be used for live imaging of transcription in a range of *Drosophila* tissues.

## INTRODUCTION

Gene expression underlies every aspect of development and homeostasis of an organism. The expression of genes at particular times or in specific cells is exquisitely controlled to ensure correct development. Transcriptional control is mediated at the level of DNA accessibility, transcription factor recruitment and RNA polymerase II (Pol II) initiation, elongation and termination (Cramer, 2019; Haberle and Stark, 2018; Hager et al., 2009; van Steensel and Furlong, 2019). Many tools have been developed to visualise and measure mRNAs within cells, including *in situ* hybridisation-based methods and genomics approaches such as RNA sequencing (Pichon et al., 2018; Stark et al., 2019). In addition, the development of live-imaging techniques allows transcription to be visualised within cells at active gene loci (Ferraro et al., 2016; Pichon et al., 2018).

The bacteriophage-derived RNA stem-loops MS2 and PP7 are used to study nascent transcription and mRNA localisation in living cells (Bertrand et al., 1998; Chubb et al., 2006; Larson et al., 2011). Multiple copies of these loops are inserted into the gene of interest using CRISPR or transgenesis. When the gene is transcribed by Pol II, the RNA stem-loop structures are formed and bound by a co-expressed fluorescently tagged coat protein, MS2 coat protein (MCP) or PP7 coat protein (PCP) (Larson et al., 2011). This allows the fluorescent signal from each transcription site to be visualised and quantitated over time, with the signal proportional to the number of Pol II molecules transcribing the gene. Live imaging has revealed bursts of transcriptional activity that can be described by a two-state promoter model, in which the promoter switches between active (ON) and inactive (OFF) states (Lionnet and Singer, 2012). Control of the promoter states is tuned by transcription factor inputs and is important for developmental processes (Garcia et al., 2020).

As a premier model organism, *Drosophila* has many genetic tools available to the scientific community. The collection of Minos-mediated integration cassette (MiMIC) insertions are stocks containing transposable elements with inverted ΦC31 recombinase target sites that are spread across the *Drosophila* genome and can be used for gene tagging via cassette exchange (Nagarkar-Jaiswal et al., 2015a,b; Venken et al., 2011). Currently, there are 7451 insertion stocks available at the Bloomington *Drosophila* Stock Center (BDSC) associated with 4367 distinct genes, which can be targeted using this technology. MiMIC insertions have an advantage over other types of genomic inserted elements (pBac and P-elements) as they have no sequence bias and can insert in any genomic location. Therefore, the insertion collection targets non-coding regions of genes, including the UTRs and intronic sequences (Venken et al., 2011). MiMIC insertions have been genetically manipulated to tag genes with various sequences to study protein expression and localisation (Nagarkar-Jaiswal et al., 2015a,b). This is achieved by recombination-mediated cassette exchange (RMCE) at the MiMIC sites within introns, without the need for microinjection. As a result, this approach enables gene tagging, as crossing two fly lines to genetically manipulate a gene region is much easier than having to design cloning strategies and perform microinjections to achieve the same outcome.

Here, we describe the generation of a new set of fly stocks that can be used to insert a variety of MS2/PP7 stem-loops into any of the thousands of genes within the existing MiMIC library. We have also made seven new fluorescent coat protein stocks that improve detection options. We validate the approach by inserting different MS2/PP7 sequences into protein-coding genes and the *roX1* long non-coding RNA (lncRNA) to study their transcription dynamics in living embryos. We have also used our new lines to extend live transcription imaging to other tissues during development, including the ovary, wing disc and larval brain. Overall, as the MiMIC-based approach relies on a simple crossing scheme, the new stocks we have generated will greatly facilitate the study of transcription of endogenous genes in living cells *in vivo*.

## RESULTS

### A new MS2/PP7 toolkit for MiMIC insertion and transcription live imaging

**Fig. 1.**
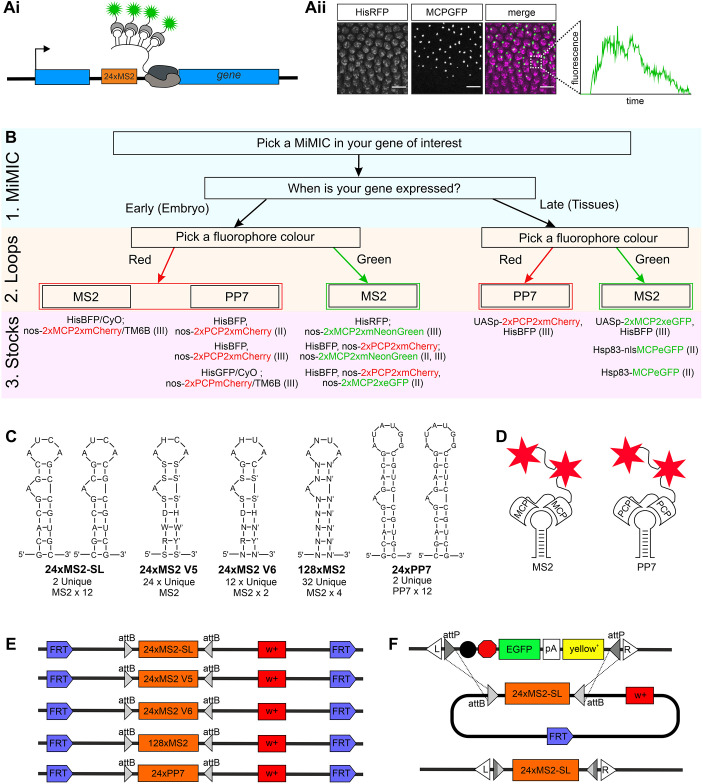
**Overview of the MiMIC insertion strategy for MS2/PP7 sequences.** (Ai) Schematic shows 24×MS2 loops (orange box) inserted into a gene intron. Following transcription, fluorescent coat proteins (grey crescents with green stars) bind to the stem-loops in the RNA. (Aii) Still from a live-imaging movie showing TSs, visualised as bright fluorescent foci of MCPGFP bound to the stem-loops, within the nuclei marked by HisRFP. Fluorescent intensity can be tracked over time as shown for the TS in one nucleus (inset). Scale bars: 10 µm. (B) A flowchart showing the three genetic components required for visualisation of live transcription *in vivo*: (1) the MiMIC insertion, (2) the stem-loop options and (3) the corresponding coat protein fly stocks generated or used in this study. *Hsp83-nlsMCPeGFP* is from Forrest and Gavis (2003). (C) Nucleotide sequences of the MS2 and PP7 loop versions used in this study. (D) Schematics of MCP or PCP bound to an MS2 or PP7 loop, respectively. In each case, a tandem dimer coat protein is fused to two fluorescent proteins (red stars). (E) Schematics of the plasmids injected into flies to create the loop donor fly stocks. (F) Scheme for insertion of the loop donor cassette into the MiMIC-containing locus. FLP/FRT-mediated removal of the cassette is followed by RMCE at the attB/attP sites within the donor and target site, mediated by germline expression of the ΦC31 integrase (top). This results in integration of the loops into the MiMIC site within the gene (bottom). Ai and Aii are adapted from Forbes Beadle et al. (2023).

Live imaging of nascent transcription requires the insertion of an array of stem-loop hairpin sequences (MS2 or PP7) into the gene of interest. These hairpins are bound by MCP or PCP, respectively, expressed as a fluorescent protein fusion (Fig. 1Ai). Transcription sites (TSs), which are visible as bright fluorescent foci within each nucleus, can be tracked over developmental time to reveal transcription dynamics (Fig. 1Aii). To facilitate live-imaging experiments, we have generated new stem-loop donor fly stocks compatible with the MiMIC system, which has a library of insertion sites in thousands of genes. We have also made new coat protein stocks. The workflow involves selection of first a MiMIC insertion in the gene of interest, second the type of loop and third the fluorescent coat protein stock (Fig. 1B).

We generated several fly lines that carry insertions with different versions of the MS2 or PP7 loops (Fig. 1C), including 24×MS2-SL, 24×MS2V5, 24×MS2V6, 128×MS2, 24×PP7 (Bertrand et al., 1998; Fukaya et al., 2016; Tantale et al., 2016; Tutucci et al., 2017). The version of the MS2 loops that was optimised for stable expression in bacteria (MS2-SL) has been used in many live-imaging studies in the *Drosophila* embryo (Fukaya et al., 2016; Garcia et al., 2013; Hoppe et al., 2020; Lammers et al., 2020; Lim et al., 2018). MS2-SL consists of 12 pairs of repetitive sequences that form stem-loop structures with short linker sequences in between. MS2V5 is an array of 24 non-repetitive stem-loop sequences (Tutucci et al., 2017). The MS2V6 variant contains a U instead of a C at a crucial nucleotide in the coat protein binding site that makes the interaction weaker and the loop–coat protein interaction less stable (Tutucci et al., 2017). A strong coat protein–loop interaction has been shown to interfere with mRNA degradation in yeast cells when the loops are in the 3′ UTR (Garcia and Parker, 2015). Although the effect of stem-loops on mRNA degradation has not been addressed in *Drosophila*, the V6 version may be useful for studies in which correct mRNA degradation of the target is important. The 128×MS2 loop variant increases the number of loops, which improves the detection and signal-to-noise ratio, allowing weak TSs and single mRNAs to be detected (Dufourt et al., 2021; Tantale et al., 2016; Vinter et al., 2021). 24×PP7 is a different stem-loop structure that can be utilised in combination with MS2 to allow dual colour imaging of each allele of the same gene or two different RNAs.

Once the stem-loops have been transcribed by Pol II, two MS2 or PP7 coat proteins bind to each loop (Fig. 1D). Expressing the coat proteins as a tandem dimer improves complete occupancy at all stem-loops and quantitation of the fluorescent signals (Wu et al., 2012). Therefore, we have generated new stocks with MCP/PCP tandem dimers fused to two fluorescent proteins to maintain the ratio of two fluorescent proteins for each stem-loop (Fig. 1D). MCP/PCP have been fused to different fluorescent proteins and the new lines typically also carry *HiseBFP2* (listed in Fig. 1B) to simultaneously visualise nuclei during imaging.

### Overview of the protocol for inserting loops into MiMIC-containing genes

We utilised the previously described genetic strategy to design the MS2/PP7 loop lines so that they can be used with RMCE to genetically insert the loop-array sequences into MiMIC-containing genes (Nagarkar-Jaiswal et al., 2015a). The MS2/PP7 loop-array cassettes were cloned between inverted attB sites (Fig. 1E), which lie upstream of a *mini-white* (*w^+^*) marker that is used for selection and to follow inheritance of the cassette. The entire cassette is flanked by FRT sites, allowing FLP-mediated excision. As our constructs are for visualising nascent transcription, these donor lines do not need splice acceptor or donor sites, or need to be in-frame, unlike previous studies that used MiMIC insertions for protein localisation (Nagarkar-Jaiswal et al., 2015a). Using a crossing scheme and heat shock, RMCE inserts the repetitive loop sequences into the gene of interest with the action of germline-expressed *vasa* ΦC31 integrase at the inverted attP sites (Fig. 1F).

**Fig. 2.**
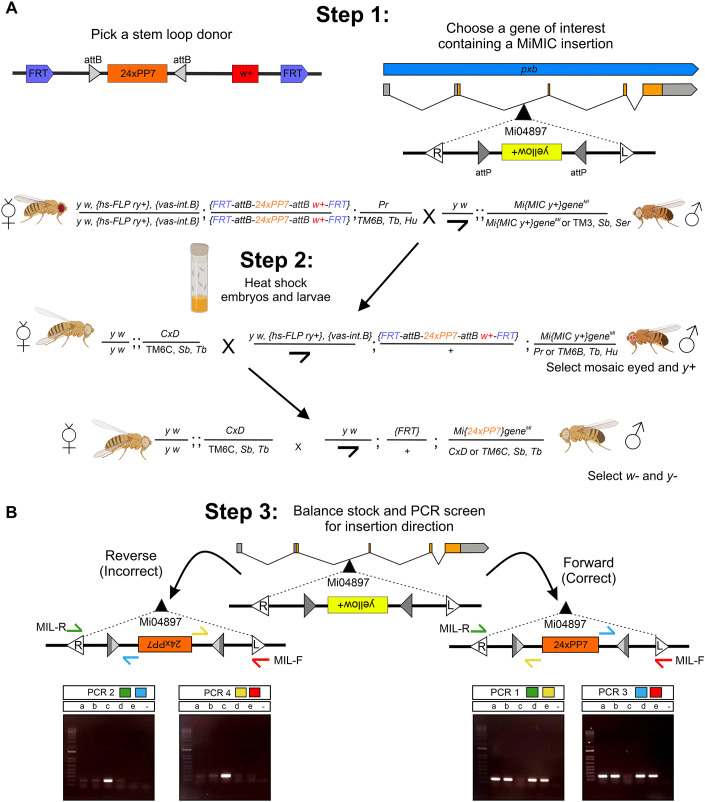
**Step-by-step protocol for inserting loops into MiMIC-containing genes.** (A) Crossing scheme to target a MiMIC-containing gene on the third chromosome (*pxb*) with 24×PP7 loops. The *pxb* gene contains an intronic MiMIC insertion (Mi04897). The Mi04897 insertion is in the opposite orientation to *pxb* and is marked by the *yellow^+^* marker and flanked by inverted attP sites (grey triangles). The selection of markers as indicated will result in flies with loops inserted into the MiMIC. (B) PCR screening of 24×PP7 loops inserted into *pxb* at Mi04897. The opposite direction relative to the gene is shown by a band in PCR2 (green and blue primers) and PCR 4 (yellow and red primers) and the absence of a band in PCR1 (green and yellow primers) and PCR 3 (blue and red primers). The same direction relative to the gene is confirmed by a band in PCR1 and PCR 3 and the absence of a band in PCR2 and PCR 4. Samples a, b, d and e here are in the correct forward orientation. MIL-F (red primer) and MIL-R (green primer) are common to all MiMIC inserts and the internal yellow and blue primers are stem-loop specific. Fly cartoons were created in BioRender. Ashe, H. (2024) https://BioRender.com/o52d992.

A simple three-step method is used to insert the loop cassette sequence into the MiMIC insertion site of a gene of interest (Fig. 2A). MiMIC insertions are readily available from the BDSC and all the stocks described here will also be made freely available. Step 1 is the selection of the loop donor and the gene of interest containing a MiMIC insertion. In this example, we inserted 24×PP7 loops into the second intron of the *pxb* gene using the Mi04897 MiMIC insertion by crossing virgin females from the PP7 stem-loop donor flies to males with the MiMIC insertion. As the heat shock-inducible FLP recombinase is more active at higher temperatures, it is important that the loop donor stocks are routinely maintained at 18°C. However, these stocks can still occasionally lose the loop donor insertion flanked by FRT sites (Nagarkar-Jaiswal et al., 2015a). Therefore, the stocks should be screened regularly to remove any individuals that have lost the *w*^+^ marker. Alternatively, the loop donor insertion stocks can be maintained without the FLP recombinase, which can be crossed in at the start of each experiment (Fig. S1A).

At step 2, the F1 embryos and larvae are heat-shocked, which liberates the loop cassette. Activation of the heat shock FLP recombinase results in mosaic-eyed progeny, which should be selected for in the emerging adults. The loop cassette can then insert into the region between the inverted attP sites of the MiMIC locus via RMCE, which results in loss of the MiMIC's *yellow*^+^ (*y*^+^) marker in the next generation.

After the stock has been established in step 3, the male parent is sacrificed for PCR screening and/or sequencing to determine the orientation of the stem-loop insert (Fig. 2B). All *yellow^−^ white^−^* (*y^−^w^−^*) males will have an insertion event, but, as the cassette can insert in either direction, we recommend that at least five to ten *yw* males are screened to obtain an insertion in the correct orientation. A combination of PCR primers can be used to determine the orientation of the loops, one overlapping the Minos L or R sequences and the other in a small intervening sequence between the attR sites and the repetitive stem-loops of the insert. In the example shown, sample c with a PCR product for primer sets PCR 2 and PCR4 had the PP7 loops inserted backwards relative to the *pxb* gene, whereas samples a, b, d and e with a PCR product for primer sets PCR1 and PCR3 had the insertion in the same orientation as the gene (Fig. 2B). Dual testing of the direction of the insert will give confidence that the loops are inserted in the correct orientation to be bound by the fluorescent coat proteins for visualisation (Valegård et al., 1994). Additional sequencing of the PCR product can be used to confirm the correct direction of the loops.

In summary, this crossing scheme is easy and efficient so that lines with integrated MS2/PP7 sequences integrated into the gene of interest can be obtained in as little as a month. The crossing schemes for insertion of a loop cassette into the X and second chromosome are shown in Fig. S1B,C. Considerations for targeting a gene on the X chromosome are discussed in the Materials and Methods. Once the desired stock has been generated, it can be crossed to fluorescent coat protein lines to visualise live transcription *in vivo*. We find no evidence that the insertion of these loops interferes with the endogenous expression pattern of any of the genes we have investigated to date, including those in this study (Fig. S2).

### Live imaging of transcription of both *pxb* alleles in the *Drosophila* embryo

To show the utility of the MiMIC-tagging live-imaging system, we used the new lines we generated that contain either 24×MS2V6 loops or 24×PP7 loops in the *pxb* locus (Fig. 2) to visualise nascent transcription in the early *Drosophila* embryo. *HisRFP; nos-2×MCP2×mNeonGreen* females lay embryos with these proteins maternally loaded. HisRFP is a nuclear marker (Schuh et al., 2007). The 2×MCP2×mNeonGreen fluorescent coat protein is a tandem MCP dimer fused to two mNeonGreen fluorescent proteins, which maintains the ratio of two fluorescent proteins bound for each stem-loop. *2×MCP2×mNeonGreen* is expressed as a transgene under the control of the *nanos* (*nos*) promoter, resulting in maternal loading of the protein. This allows time for the fluorescent protein to mature by the time zygotic genes are transcribed in the early embryo.

**Fig. 3.**
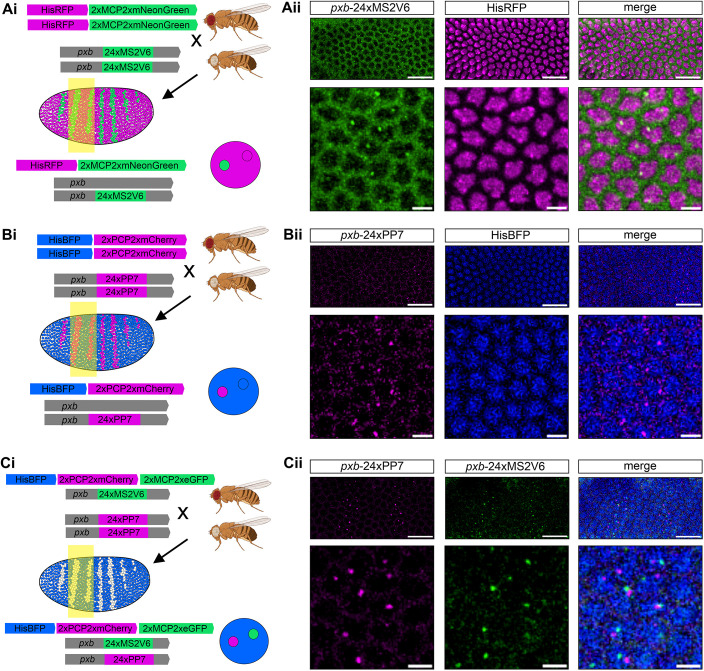
**Transcription live imaging of *pxb* tagged with MS2 and PP7 stem-loops in embryos.** (Ai) Schematic showing the *pxb* gene locus with the 24×MS2V6 insertion. Males with the 24×MS2V6 loops inserted into *pxb* are crossed to females expressing *HisRFP* and *nos-2×MCP2×mNeonGreen*. Embryos have the *pxb-24×MS2V6* TS visible as an intense mNeonGreen signal within the HisRFP nucleus; the imaging region is shown in yellow. (Aii) Top: still images from a time-lapse movie of *pxb-24×MS2V6* TSs in two anterior stripes in the nc14 embryo. Scale bars: 20 µm. Bottom: higher magnification images from nuclei with the stripe. Nuclei are marked in magenta and *pxb-MS2* TSs are in green. Scale bars: 5 µm. (Bi) As in Ai but with 24×PP7 loops inserted into *pxb* and females are expressing *HiseBFP2* and *nos-2×PCP2×mCherry*. (Bii) Still from a movie of an embryo expressing *HiseBFP2* and *nos-2×PCP2×mCherry* marking the nuclei in blue and the *pxb-PP7* TSs in magenta. (Ci) Schematic showing *pxb* labelled to visualise each allele with a different loop type (MS2 or PP7). (Cii) Dual imaging of these embryos detects transcription of both alleles. *pxb-PP7* TSs are in magenta, *pxb-MS2* TSs are in green, nuclei are blue in the merge. See also Movies 1-3. Fly cartoons created in BioRender. Ashe, H. (2024) https://BioRender.com/u96a435.

After crossing *HisRFP; nos-2×MCP2×mNeonGreen* females to *pxb-24×MS2V6* males, live imaging of the resulting embryos detected RFP-labelled nuclei and fluorescent *pxb* TS foci labelled with mNeonGreen (Fig. 3Ai). Only one of the two *pxb* alleles was tagged by 24×MS2V6 in the embryo (the one crossed in from the male). Live imaging of embryos towards the end of nuclear cycle 14 allowed visualisation of the TSs within the nuclei of cells within the *pxb* expression domain (Fig. 3Aii, Movie 1). The endogenous pattern of *pxb* was striped across the embryo at this stage (Inaki et al., 2002); only two of the anterior stripes of the expression domain are shown in the movie still (Fig. 3Aii), as highlighted by the yellow region on the schematic (Fig. 3Ai). The TSs detected were consistent with the endogenous expression of *pxb* within this region (Fig. S2). In control embryos from *HisRFP; nos-2×MCP2×mNeonGreen* virgin females crossed to wild-type males (Fig. S3Ai, Movie 4), no intense *pxb* transcription foci in the absence of the loops inserted into *pxb* were observed (Fig. S3Aii). However, we did detect weaker fluorescent puncta in the apical region of all nuclei, which may relate to expression of tdMCP (Fig. S3D; see Discussion).

Next, we tested the *pxb-24×PP7* insertion by crossing males to females expressing *HiseBFP2* and *nos-2×PCP2×mCherry* (Fig. 3Bi). Imaging detected mCherry fluorescent *pxb* TS foci within BFP-labelled nuclei (Fig. 3Bii), which were absent from embryos laid from females expressing *HiseBFP2* and *nos-2×PCP2×mCherry* crossed to wild-type males (Fig. S3Bi,Bii, Movie 5). The *pxb-24×PP7* embryos showed the same expression pattern in the anterior stripes as described for the *pxb-24×MS2* embryos (Fig. 3Bii, Movie 2).

As two different types of loops were inserted in the *pxb* locus, we crossed the *pxb-24×MS2* and *pxb-24×PP7* lines together to visualise both alleles at the same time (Fig. 3Ci). Females heterozygous for the *HiseBFP2*, *nos-2×PCP2×mCherry*, *nos-2×MCP2×eGFP* and *pxb-24×MS2* insertions were crossed to *pxb-24×PP7* homozygous males, allowing visualisation of both *pxb* alleles in half of the resulting embryos (Fig. 3Cii, Movie 3). Although we depict the embryo inheriting the *HiseBFP2*, *nos-2×PCP2×mCherry* and *nos-2×MCP2×eGFP* transgenes in Fig. 3Ci, transcription can be visualised in embryos lacking these transgenes as the *HiseBFP2* and fluorescent coat proteins are maternally deposited in the egg. We observed cell-to-cell variation in the signal from each allele (Fig. 3Cii), consistent with transcriptional bursting. Although bright transcription foci were visible in a striped pattern, again there was some weaker ubiquitous 2×MCP2×eGFP background; this was distinguishable from the TS signals as they were observed in different *z*-slices (Fig. S3Ci,Cii,E). The control embryo shown in Fig. S3Cii,E and Movie 6 was obtained from a female homozygous for the *HiseBFP2*, *nos-2×PCP2×mCherry* and *nos-2×MCP2×eGFP* transgenes, whereas the embryo in Fig. 3Cii was obtained from a heterozygous female. For direct comparisons of TSs and background puncta for the MCP lines, see Fig. S3D,E. We also note that control embryos laid from *nos-2×MCP2×eGFP* homozygous mothers formed larger MCPeGFP aggregates at later stages during gastrulation (Fig. S3F, Movie 6), so this line may not be suitable for target genes expressed in gastrulating embryos. Nonetheless, the new stocks we have generated allow *pxb* transcription to be visualised in the early embryo. Analysis of transcription of both alleles can be used to infer burst parameters and study the degree of coordination of transcription between both alleles as well as transcriptional heterogeneity.

### Live imaging of transcription of the BMP target gene *Race* and *roX1* lncRNA

**Fig. 4.**
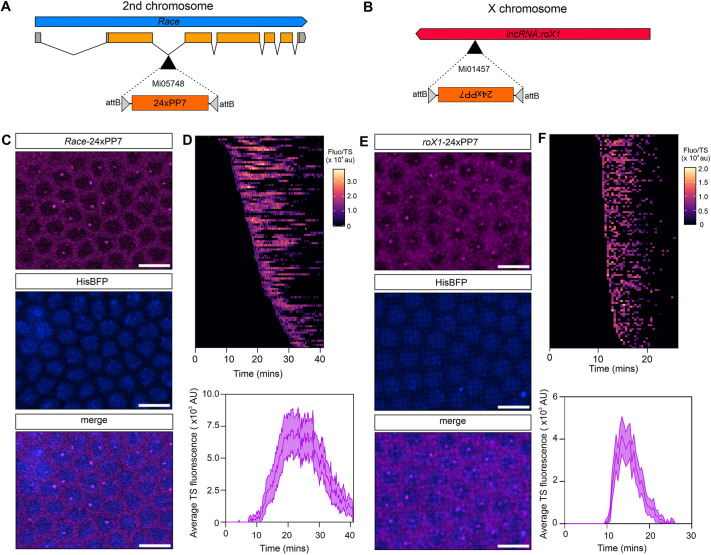
**Live imaging of *Race* and *roX1* transcription.** (A,B) 24×PP7 loops were inserted into the second chromosome gene *Race* at Mi05748 (A) and the X chromosome lncRNA *roX1* at Mi01457 (B). (C) Stills from a live-imaging movie of an embryo with *Race-24×PP7* and maternally expressed *HiseBFP2* and *nos-2×PCP2×mCherry* under the control of the *nos* promoter, showing nascent TSs within the *Race* expression domain. (D) Top: heatmap of individual *Race* transcription site traces measured during nc14. Bottom: graph showing mean TS fluorescence intensity of *Race* in a nc14 embryo. (E,F) As in C,D, but the data are shown for *roX1-24×PP7*. Mean±CI for 72 nuclei (*Race*) and 115 nuclei (*roX1*). Scale bars: 20 µm.

We next targeted the other major chromosomes in *Drosophila* and inserted loops into genes on the X and second chromosomes. For the second chromosome, we chose the Dpp target gene *Race* (also known as *Ance*) (Ashe and Levine, 1999; Tatei et al., 1995). *Race* contains a MiMIC insertion, Mi05748, within the second intron (Fig. 4A). We used this MiMIC insertion to insert 24×PP7 loops into the endogenous locus as described in Fig. 2 using the crossing scheme in Fig. S1C. This 24×PP7 insertion tags all *Race* isoforms. For the X chromosome, we used Mi01457 to insert 24×PP7 loops to tag three of the five isoforms of the *RNA on the X 1* (*roX1*) lncRNA, which plays a crucial role in dosage compensation in males (Samata and Akhtar, 2018) (Fig. 4B).

Females expressing *HiseBFP2* and *nos-2×PCP2×mCherry* were crossed to *Race-24×PP7* males and the resulting embryos were imaged during nuclear cycle (nc) 14 (Movie 7). A representative image of the anterior dorsal view of the embryo shows nuclei transcribing *Race* during this time (Fig. 4C). *Race* TSs were tracked and the single-nuclear transcriptional fluorescent traces show stochastic onset in nc14 (Fig. 4D). Calculating the mean TS fluorescence showed that transcription peaks ∼20 min into nc14 (Fig. 4D).

*roX1-24×PP7*; *HiseBFP2*, *nos-2×PCP2×mCherry* females were crossed to *roX1-24×PP7* males and embryos collected to detect *roX1* transcription across early development. Ubiquitous transcription of this lncRNA was observed during nc14 (Fig. 4E). Transcription initiates synchronously and was detected in a short time period from approximately 10-25 min into nc14 (Fig. 4F, Movie 8). This timing is consistent with the previously described *roX1* expression in embryos (Meller et al., 1997). Together, these experiments and those shown for *pxb* validate that we can use the MiMIC approach to target MS2/PP7 loops into the three major chromosomes in *Drosophila.* This has allowed us to visualise and track transcriptional activity of, not just mRNAs, but also a lncRNA, which offers new possibilities for studying non-coding RNA (ncRNA) transcriptional dynamics.

### Live imaging of transcription in the ovary, wing disc and larval brain

We next addressed whether the new stem-loop lines we have generated could be used to visualise nascent transcription in other tissues later in development. To this end, we constructed new fly stocks for expression of the fluorescent coat proteins. We first tested the GAL4-UAS system (Brand and Perrimon, 1993) and tried different versions of the upstream activating sequence (UAS) to drive varying levels of expression in somatic and germline tissues. UASt is suitable for use in somatic tissues but has poor expression in the germline. UASp, however, overcomes this limitation and drives stronger expression in the germline relative to somatic tissues (DeLuca and Spradling, 2018). As we wanted our tools to target both somatic and germline tissues, we made UASt (Brand and Perrimon, 1993) and UASp (Rørth, 1998) versions of 2×MCP2×eGFP and 2×PCP2×mCherry. However, we found that expressing *UASt-2×MCP2×eGFP* or *UASt-2×PCP2×mCherry* in somatic tissues, such as the wing disc, with GAL4 drivers resulted in very high expression levels that caused accumulation of excess coat protein fusions in intense aggregates. As a result, TSs were undetectable (Fig. S5F). Therefore, we next tried using UASp to drive expression in the somatic tissues using the same drivers, as UASp transgenes have a much lower level of expression in somatic tissues compared to UASt transgenes (DeLuca and Spradling, 2018). We found that by using *UASp-2×MCP2×eGFP*, expression was lower in the somatic tissues we tested including the wing disc, brain and ovarian follicle cells. As this avoided accumulation of the strongly fluorescent MCPeGFP aggregates, we used these transgenes in our further experiments.

**Fig. 5.**
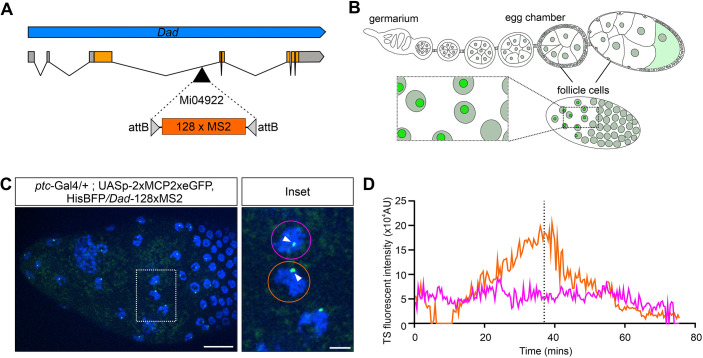
**Visualising nascent transcription in the ovary.** (A) Schematic of the 128×MS2 loop array inserted into the Mi04922 insertion in the first intron of the *Dad* gene. (B) Schematic showing the female ovary and the follicle cells that surround the egg chambers and oocyte. (C) Still from a live-imaging movie of a stage 9 egg chamber from a *ptc-Gal4/+; UASp-2×MCP2×eGFP, HiseBFP2/Dad-128×MS2* female, anterior is to the left. *Dad-128×MS2* TSs are detected in the anterior follicle cells with a region of expressing cells marked by the white box. Scale bar: 20 µm. Inset: a higher magnification image of the follicle cells highlighting two cells (magenta and orange circles) with TSs (arrowheads). Scale bar: 5 µm. (D) Fluorescence intensity traces from the two cells highlighted in the inset. The colours denote the different cells in the inset in C.

We used a fly line with *ptc-Gal4* driving *UASp-2×MCP2×eGFP* to visualise MS2 transcription in larval and adult tissues. *ptc-Gal4* is expressed in the wing disc, follicle cells and brain (Weaver et al., 2020). We focussed on the *Daughters against decapentaplegic* (*Dad*) gene, which is expressed in the ovary and brain, and studied *Dad* transcription in these tissues by using a MiMIC insertion to create a fly line with 128×MS2 loops in the third intron (Fig. 5A). Here, we tested 128×MS2 loops as the Mi04922 insertion within the intron of *Dad* is close to the next exon (∼1.1 kb away). We hypothesised that the larger number of stem-loops would give a stronger signal with more fluorescent MCP bound compared to the 24×MS2 before the intron is spliced, but have not generated *Dad-24×MS2* to test this directly. *ptc-Gal4*; *UASp-2×MCP2×eGFP*, *HiseBFP2* females were crossed to *Dad-128×MS2* males. In dissected tissue from the female progeny, we live imaged the follicle cells (Fig. 5B) and detected transcription of *Dad-128×MS2* in the anterior follicle cells of stage 9 egg chambers (Fig. 5C, Movie 9). This is consistent with previous data using a *Dad-lacZ* reporter line showing expression in anterior follicle cells from stage 8 to stage 10 egg chambers (Shravage et al., 2007). Moreover, visualisation of nascent TSs in these tissues using single-molecule fluorescence *in situ* hybridisation (smFISH) with endogenous *Dad* probes and MS2 probes confirmed that MS2 detection accurately reflects the endogenous transcription of this gene in stage 8 egg chambers (Fig. S4A,B).

Live imaging over a period of an hour showed that transcription was maintained in the tissue throughout the imaging period. Transcriptional traces of two cells showed that they had different active transcription times and off periods (Fig. 5D). The cell outlined in magenta in Fig. 5C maintained a stable level of transcription throughout the imaging window, whereas the cell outlined in orange had a period during which transcription was off for ∼5 min and then had a higher level of transcription between 20 and 45 min compared to the magenta cell (Fig. 5D). These data demonstrate that transcription can be imaged in ovarian follicle cells over a significant time and suggest that there is heterogeneity in the transcriptional activity between cells.

**Fig. 6.**
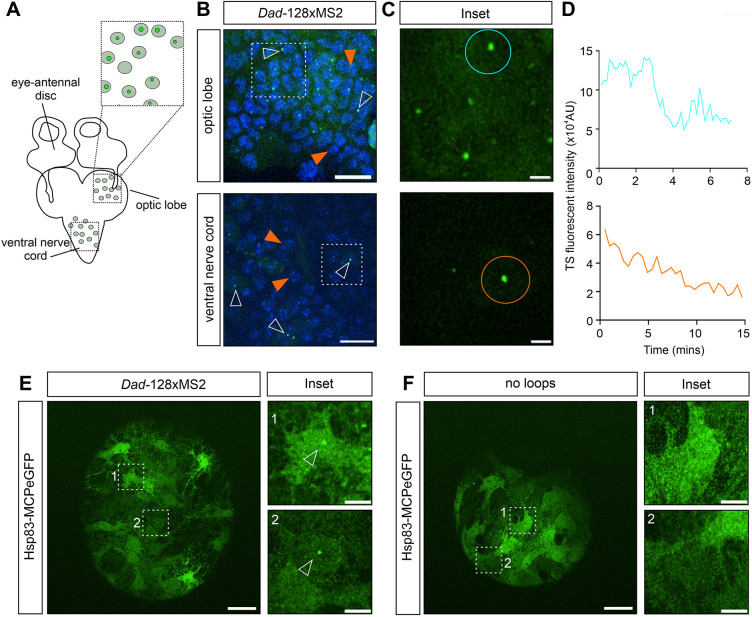
**Visualising nascent transcription in the larval brain.** (A) Schematic showing the third instar larval brain and eye-antennal discs highlighting the regions of the optic lobe and ventral nerve cord that were imaged live. (B) Stills from live-imaging movies of cells from *ptc-Gal4*; *UASp-2×MCP2×eGFP*, *HiseBFP2* and *Dad-128×MS2* larval brains. A number of cells have active TSs (unfilled arrowheads), and examples of non-transcribing cells are also present (orange arrowheads). Scale bars: 10 µm. (C) Higher magnification of the outlined regions in B. Scale bars: 2 µm. (D) Transcription traces from the circled brain cells in C. (E) Still from a live-imaging movie of glial cells from *Hsp83-MCPeGFP* and *Dad-128×MS2* larval brains. Scale bar: 20 µm. Higher magnification of two cells with a *Dad-128×MS2* TS is shown in the inset to the right. Scale bars: 5 µm. (F) As in E but larval brains are from *Hsp83-MCPeGFP/+* individuals with no MS2 loops in *Dad*.

We next live-imaged dissected third instar larval brains (Fig. 6A) and observed *Dad-128×MS2* TSs in cells of the optic lobe and ventral nerve cord (Fig. 6B,C, Movie 10). Transcription of *Dad* in the larval brain is consistent with previous reports of a *Dad-lacZ* reporter expressed in the ventral nerve cord (Vuilleumier et al., 2019). Tracking of TSs within these cells showed dynamic transcription across a 15-min imaging period (Fig. 6D). Together, these data show the feasibility of studying transcription dynamics over varying time periods of minutes up to hours in living tissues of the larva and adult fly.

In addition to the UASp lines, we also tested an existing stock with the *Hsp83* promoter driving expression of MCPeGFP with a nuclear localisation signal (Forrest and Gavis, 2003) (referred to here as *Hsp83-nlsMCPeGFP*) to visualise MS2-tagged mRNAs in larval and adult tissues. *Dad-128×MS2* males were crossed to *Hsp83-nlsMCPeGFP* females to detect transcription of *Dad-128×MS2* in the cells of the ovary (Fig. S4C,D) and wing disc (Fig. S5). We again detected *Dad-128×MS2* TSs in the anterior follicle cells of the ovary, as observed with *ptc-Gal4*; *UASp-2×MCP2×eGFP*, and tracked these nuclei for more than 2 h (Fig. S4C,D).

Live imaging of *Dad-128×MS2* in the wing disc focussed on the cells of the peripodial membrane on the surface of the wing disc pouch (Fig. S5A). TSs, with stronger signals than the low level of free MCPeGFP in nuclei, were observed in many cells (Fig. S5Bi) and this expression was confirmed in fixed tissue using smFISH (Fig. S5D). In addition, we observed nonspecific accumulation of MCPeGFP in a larger structure within the nucleus (Fig. S5Bii), which appears to be the nucleolus based on colocalisation of the GFP signal with staining for the nucleolar marker Fibrillarin in fixed wing disc tissue (Fig. S5E). These structures were less obvious when TSs were present. Therefore, it will be important to include a control without MS2 if using the *Hsp83-nlsMCPeGFP* stock, to confirm that the transcription site is being labelled in addition to the nucleolus (Fig. S5D). We rarely observed the nascent transcription site localised near to the nucleolar region and were able to track the TSs in individual nuclei over time, which showed bursts of transcription (Fig. S5C).

Due to the non-specific nuclear accumulation observed using the existing *Hsp83-nlsMCPeGFP* stock, we made a new line to ubiquitously express MCPeGFP in which the nuclear localisation signal was removed: *Hsp83-MCPeGFP*. *Hsp83* is expressed in many tissues even in the absence of heat shock, so we reasoned that it was a good candidate for semi-ubiquitous expression (Mason et al., 1984; Xiao and Lis, 1989). We tested this new stock by crossing *Hsp83-MCPeGFP* females to *Dad-128×MS2* males and dissecting brains from the *Hsp83-MCPeGFP/+*; *Dad-128×MS2/+* larvae. We observed *Dad* TSs in the perineural glial cells of the brain (Fig. 6E), but not in brains from *Hsp83-MCPeGFP/+* control larvae without *Dad-128×MS2* (Fig. 6F). In addition, no nucleolar accumulation of MCPeGFP was observed. Expression in the perineural glial cells has been previously reported for the *Dad-lacZ* reporter (Kanai et al., 2018).

Although the *Hsp83* promoter has previously been reported to be a ubiquitous promoter, we observed different MCPeGFP expression levels between cells (see highlighted cells in Fig. 6E,F). Therefore, various MCP-expressing lines should be tested for the specific tissue of interest and our additional stocks (*Hsp83*, *nos*, UASt, UASp) will facilitate this. Overall, these data show that the MiMIC-based approach can be used to insert MS2/PP7 loops into endogenous genes, facilitating the visualisation of transcription live for long periods of time in different *Drosophila* tissues.

## DISCUSSION

Here, we have developed a new set of *Drosophila* stocks that allow MS2/PP7 stem-loops to be inserted into protein-coding genes and ncRNAs to visualise nascent transcription *in vivo*. We have exploited a previously reported MiMIC-based gene-tagging strategy that relies on RMCE (Nagarkar-Jaiswal et al., 2015a) and combined it with our new stocks so that insertion of the stem-loops relies only on a simple crossing scheme and PCR screening. As proof of principle, we have used different MiMIC stocks to show how this approach can be used to visualise transcription of different genes and a lncRNA in distinct tissues including the embryo, larval wing disc, brain and ovary. The new coat protein lines and the ease of use of this MiMIC-based approach will benefit many researchers in the *Drosophila* research community.

Current methods to insert MS2 or PP7 loops into endogenous genes using CRISPR can be time-consuming and difficult. CRISPR requires many cloning steps or the design plus synthesis of gene strings, followed by embryo microinjections, screening for successful CRISPR events, verification of the insertion and establishment of the stocks. Approximately 10 weeks is needed for stem-loops to be inserted into a gene locus (Gratz et al., 2015; Hoppe and Ashe, 2021a; Yu et al., 2021). Although transgenic lines can be more rapidly generated, these may not contain all the regulatory sequences required to fully recapitulate the endogenous transcription dynamics. The method presented here can routinely be performed in ∼5 weeks as the process simply involves crossing fly stocks followed by verification of the insertion by PCR, while avoiding the more costly and time-consuming steps of cloning and microinjection.

Combining these new reagents with the MiMIC insertion collection offers the opportunity to target at least 4367 genes throughout the genome. One obvious limitation to the system presented here is that a MiMIC insertion is required. If the specific gene of interest does not contain a MiMIC insertion, then a CRiMIC insertion could be generated. CRiMIC insertions are MiMIC insertions inserted into the genome via CRISPR mutagenesis so that they are targeted to genes rather than being randomly inserted (Lee et al., 2018). Generating a CRiMIC has the advantage that it would also allow fluorescent protein tagging in addition to insertion of stem-loops (Lee et al., 2018). Many CRiMIC insertions are available from the BDSC, with the number likely to rise in the future.

One benefit of utilising the MiMIC-based approach described here is that there is an abundance of target sites located in the UTRs and introns of genes, which are the preferred sites of MS2/PP7 stem-loop insertions to avoid disrupting coding sequences. MS2/PP7 stem-loops inserted in the 5′ UTR, 3′ UTR or an intron have all been used to study transcription dynamics previously (Forbes Beadle et al., 2023; Garcia et al., 2013; Lucas et al., 2013; Whitney et al., 2022). Each position has advantages and disadvantages relating to the study of promoter states (Ferraro et al., 2016). While insertion into the 5′ UTR results in a high signal to noise, the persistence of fluorescence hampers the direct detection of transient off states. Conversely, with stem-loops in the 3′ UTR the fluctuations in signal better represent the promoter states but the signal is much weaker and more sensitive to background coat protein levels. If the MS2/PP7 loops are inserted into the intron, then the fluorescent signal depends on both the promoter state and the efficiency of splicing (Ferraro et al., 2016). However, data on intron half-lives in *Drosophila* S2 cells are available, which show that the median intron half-life is only 2 min (Pai et al., 2017).

Our data demonstrate how insertion of PP7 and MS2 sequences into the same MiMIC site can be used to simultaneously visualise both *pxb* alleles in the same embryo. Transcription heterogeneity across a cell population arises from extrinsic variation, due to the cellular environment or differences in concentrations of regulatory molecules between cells, and intrinsic variation, due to the stochastic nature of transcriptional bursting (Elowitz et al., 2002; Rodriguez et al., 2019). By visualising transcription of both alleles, the degree of correlation between bursts and the contribution of extrinsic and intrinsic variation to the transcription onset and end times can be determined (Falo-Sanjuan et al., 2019; Hoppe et al., 2020). In addition to studying transcription dynamics, MS2/PP7 tagging of genes and detection of TSs can be used to mark cell populations and development events. For example, an *engrailed-MS2* reporter has been used to identify and study parasegment boundaries during germ band extension in living wild-type and mutant embryos (Sharrock et al., 2022). Finally, the 128×MS2 cassette allows single mRNAs to be visualised in the cytoplasm (Dufourt et al., 2021; Vinter et al., 2021), facilitating the study of mRNA localisation.

MiMIC-based MS2/PP7 insertions can also be used to study the transcription and localisation of ncRNAs. FlyBase reports ∼200 MiMIC-containing lncRNA stocks available across the genome. Previous studies inserted 24×MS2 loops into the *iab-8* ncRNA from the bithorax complex (Arib et al., 2015) and 6×MS2 into the *roX1* ncRNA (Apte et al., 2014). However, these stocks were only used to visualise the TSs in fixed tissues. Here, by inserting 24×PP7 sites into a MiMIC in *roX1*, we have visualised *roX1* transcription in embryos during nc14. Our data show that *roX1* transcriptional activation in individual cells is very synchronous in the embryo, and that it is only transcribed transiently. How *roX1* transcription kinetics relate to its function in dosage compensation at different times and in distinct tissues is an interesting question that could be investigated with this stock in the future. Allele-sensitive single-cell RNA sequencing has revealed that mammalian lncRNAs have a high cell-to-cell variability and lower abundance due to a reduced burst frequency compared to mRNAs in the same tissue (Johnsson et al., 2022). It will also be interesting to determine how transcriptional burst parameters from ncRNAs compare to those from mRNAs during *Drosophila* development. The new coat protein lines generated here will facilitate studies of transcription of a protein-coding gene and lncRNA in the same cells during *Drosophila* development. Additionally, MS2 insertions in lncRNAs or mRNAs can be used to identify RNA-binding proteins using RNA proximity biotinylation (RNA-BioID) (Mukherjee et al., 2019) or a yeast three-hybrid approach (Cai et al., 2024).

Tagging of ncRNAs with stem-loops must be carefully considered if the function of the RNA is of interest following visualisation of nascent transcription. ncRNAs have structural and regulatory roles (Tsagakis et al., 2020), which could theoretically be affected by loop insertion. Some lncRNAs are present in the cytoplasm where they sequester miRNAs or regulate translation (Tsagakis et al., 2020). For *roX1*, we observed TSs by inserting 24×PP7 but were unable to detect *roX1* RNAs outside of the nucleus. While 24 copies of MS2/PP7 loops is sufficient to detect signals where there are multiple lncRNAs/mRNAs, e.g. TSs, the 128×MS2 cassette facilitates detection of single RNAs in *Drosophila* (Dufourt et al., 2021; Vinter et al., 2021). However, the 128×MS2 cassette is longer (∼2.2 times longer than 24×MS2/PP7), so its insertion could potentially increase the risk of disrupting lncRNA function.

The vast majority of live transcription studies in *Drosophila* have been performed in the early embryo. Here, we have shown how MS2 imaging can be used to visualise and quantitate live transcription of the *Dad* gene in the ovary, larval brain and wing disc. In the ovary, long-term imaging of *Dad-128×MS2* TSs showed heterogeneity in the transcriptional responses in individual cells, whereas bursty *Dad* transcription was detected in wing disc cells. To express the coat proteins for live imaging in the ovary and larval brain, we found that the use of GAL4 drivers with the UASp expression system, which is weak in somatic tissues (DeLuca and Spradling, 2018), allowed the best signal-to-noise detection of TSs. In contrast, we found that the very high levels of fluorescent coat proteins obtained with the GAL4/UASt system caused accumulation in cells that impeded detection of live, nascent TS signals. We also made a *Hsp83-MCPeGFP* stock, which does not show the nucleolar accumulation observed with *Hsp83-nlsMCPeGFP*, but the MCPeGFP expression level across larval brain cells was variable. We generated tandem dimer (2×MCP) stocks to allow better loop occupancy and quantitation (Wu et al., 2012). However, we detect weak background fluorescent puncta in embryonic nuclei with nos-2×MCPeGFP and nos-2×MCPmNeonGreen fusions, possibly because the 2×MCP and/or particular fluorescent proteins are prone to aggregation (Campbell et al., 2015; Eck et al., 2024 preprint). These findings highlight the importance of testing the MCP/PCP stocks in the tissue of interest for uniform expression and low aggregation in control experiments, prior to crossing in a MS2/PP7 insertion. In addition, MS2/PP7 smFISH is useful to verify that the signals observed are TSs and not background fluorescent puncta.

As these new imaging tools allow imaging nascent transcription in various tissues, keeping tissues alive and transcriptionally active during live imaging is an important consideration. However, many protocols already exist that allow *ex vivo* imaging of tissues such as we have used here in the larval discs (Dye, 2022) and the ovary (Wilcockson and Ashe, 2021). As these tissues have longer developmental time windows than the early embryo, this will allow new features of promoter dynamics and transcription to be studied. For example, it is possible that multi-scale bursting will be observed in which the promoter fluctuates on both short (minutes) and long (hours) time scales, as has been described in human cells (Tantale et al., 2016).

In summary, the MiMIC-based approach for inserting MS2/PP7 stem-loops and new coat protein lines that we have described here will be useful to many researchers interested in probing transcriptional dynamics and/or marking specific cell types in their tissue of interest. Moreover, such studies of *in vivo* transcriptional dynamics can be combined with other techniques, such as single-cell transcriptomics, to provide wholistic and time-resolved models of transcription in varying developmental contexts and in response to different cues.

## MATERIALS AND METHODS

### Fly stocks

All stocks were routinely maintained at 18°C and experiments performed at 25°C unless otherwise specified on standard fly food media (yeast 50 g/l, glucose 78 g/l, maize 72 g/l, agar 8 g/l, 10% Nipagen in ethanol 27 ml/l and propionic acid 3 ml/l). All stocks used and produced in this study are listed in Table S3.

### Cloning

#### pW35-attB1-2 and loop donor plasmids

To construct the loop donor cassette plasmids, the pBS-KS-attB1-2-PT-SA-SD-0-2×TY1-V5 cloning plasmid (Addgene, #61255) was cut with NheI and NsiI to obtain a 264 bp fragment with the inverted attB sites, and this fragment was inserted into pW35 [*Drosophila* Genomics Resource Center (DGRC), stock 1168] that had been linearised with PstI and AvrII to form a pW35-attB1-2 plasmid. Each of the repetitive loop sequences were cut out of their original plasmids and inserted into the pW35-attB1-2 plasmid. Plasmids pCR4-24×MS2SL-stable (Addgene, #31865), pBlueScript-24×PP7 (Fukaya et al., 2016) and pET264-pUC 24×MS2V6 Loxp KANr Loxp (Addgene, #104393) were digested using BamHI and BglII to obtain 24×MS2-SL, 24×PP7 and 24×MS2V6, respectively. These fragments were inserted into the pW35-attB1-2 plasmid, which had been digested with BglII. For 24×MS2V5, the cassette was PCR amplified out of the original plasmid pUbC-FLAG-24×SuntagV4-oxEBFP-AID-baUTR1-24×MS2V5-Wpre (Addgene, #84561) using primers containing additional PstI flanking sequences, digested with PstI and inserted into pW35-attB1-2 cut with PstI. For 128×MS2 cloning, an extra MCS containing an NheI site was first cloned into pW35-attB1-2 using PstI and BamHI. The 128×MS2 loops from pMK123-128MS2(XbaI) (Tantale et al., 2016) were excised using XbaI and BamHI and inserted into the BamHI and NheI sites of pW35-attB1-2.

#### pCasper-attB-nos-2×MCP2×eGFP, pCasper-attB-nos-2×MCP2×mCherry and pCasper-attB-nos-2×PCP2×mCherry

A plasmid modified from pCaSpeR 4 (DGRC, stock 1213) with an added attB site was used to insert the 948 bp *nanos* promoter region (3R:19156372-19157319) into the EcoRI site and the 1295 bp tubulin 3′UTR and downstream genomic region (3R:7088576-7089870) in the XbaI site. This pCasper-attB-nos>tub3′UTR+1 kb plasmid (Vinter et al., 2021) was used to clone 2×MCP and 2×PCP with 2×mCherry and 2×eGFP. 2×MCP was derived from pHsp83-NLS-HA-2×MCP-2×TagRFP-T (Addgene, #71242) (Halstead et al., 2015) and cloned 5′ to 2×eGFP or 2×mCherry amplified from the pHsp83-NLS-HA-2×PCP-2×GFP (Addgene, #71243) (Halstead et al., 2015) or pTV Cherry (DGRC, stock 1338) plasmids into KpnI/BamHI of pCasper-attB-nos>tub3′UTR+1 kb, with a linker between the two GFP fluorophores using Infusion (Clontech, Takara Biosciences). 2×PCP from the pHsp83-NLS-HA-2×PCP-2×GFP plasmid (Addgene, #71243) (Halstead et al., 2015) was cloned 5′ to 2×mCherry from pTV Cherry (DGRC, stock 1338) into KpnI/BamHI of pCasper-attB-nos>tub3′UTR+1 kb, with a G_4_SG_4_S_2_RM linker between the two fluorophores using Infusion (Clontech, Takara Biosciences).

#### pCasper-attB-nos-2×MCP2×mNeonGreen

The NLS-2×MCP from pHsp83-NLS-HA-2×MCP-2×TagRFP-T (Addgene, #71242) (Halstead et al., 2015) was cloned into KpnI/SpeI of pCasper-attB-nos>tub3′UTR+1 kb. The NLS was removed from the 2×MCP by digesting with KpnI and NheI and repaired using primer annealing. 2×mNeonGreen sequences were added from pCasper-nos>scFv-mNeonGreen-GB1-NLS (Vinter et al., 2021) and inserted into the SpeI site downstream of the 2×MCP using the primers listed in Table S1.

#### pCasper-attB-nos-2×PCP2×mCherry-His2Av-eBFP2

The His2Av-eBFP2 insert from nanos>SV40NLS-mCherry-PCP, His2Av-eBFP2 (Fukaya et al., 2017) was subcloned into the SacII site of pCasper-attB-nos-2×PCP-2×mCherry.

#### pUASp-attB-2×MCP2×eGFP-His2Av-eBFP2, pUASp-attB-2×PCP-2×mCherry-His2Av-eBFP2, pUASt-attB-2×MCP2×eGFP and pUASt-attB-2×PCP-2×mCherry

The 2×PCP-2×mCherry-tubulin 3′UTR and 2×MCP2×eGFP-tubulin 3′UTR fragments were subcloned from the above pCasper-attB plasmids into KpnI/NdeI of pUASp-attB (DGRC, stock 1358) and EcoRI/NotI of pUASt-attB (DGRC, stock 1419). The His2Av-eBFP2 insert was subcloned into SacII of pUASp-attB-2×PCP2×mCherry and pUASp-attB-2×MCP2×eGFP.

#### pHsp83-MCPeGFP

Hsp83-MCP-eGFP-tubulin 3′UTR was cloned into StuI/NdeI of pUASp-attB (DGRC, stock 1358) using 3-way Infusion cloning. The eGFP-tubulin 3′UTR was subcloned from the above pCasper-attB plasmids. The *Hsp83* promoter (3L:3192345-3193225) was cloned from genomic DNA. MCP was subcloned from the above pCasper-attB plasmids.

### Microinjections and transformant screening

Plasmid DNA was prepared for microinjection using a QIAGEN Plasmid Mini or Maxiprep kit (12123 or 12163) according to the manufacturer's instructions and including an extra PB wash step. DNA was diluted to the required injection concentrations in nuclease-free water and all injections were performed by The University of Manchester or University of Cambridge fly facilities. For the loop donor cassettes (24×MS2-SL, 24×PP7, 24×MS2V5, 24×MS2V6 and 128×MS2), pCasper-nos-2×MCP2×mCherry and pCasper-nos-2×PCP2×mCherry, the plasmid DNA was injected into *w*^1118^ *Drosophila* embryos with P-element helper plasmid pUC hsPI[delta2-3] (DGRC, stock 1001) at 0.8 µg/µl and 0.5 µg/µl, respectively. Successful random P-element insertions were selected by screening for red-eyed progeny (*w*^+^) after crossing the injected individuals to *w*^1118^ flies. Transformants were crossed to a second and third chromosome balancer stock (w*; If/CyO; MKRS/TM6B) to map them to chromosomes and subsequently the P-element was mapped using inverse PCR (Bellen et al., 2004). At least one insertion on the second and third chromosome was kept for subsequent crossing. Loop-donor transformants were then crossed into the P{hsFLP}12, y^1^ w* M{vas-int.B}ZH-2A; S^1^/CyO; Pri^1^/TM6B, Tb^1^ (BDSC:33216) background and maintained at 18°C to avoid leaky expression of the hsFLP, which can occasionally remove the loop donor cassette. Escaper flies with *white*^−^ eyes, which occasionally flip out the loop cassette flanked by FRT sites, were routinely removed from stocks to maintain the loop cassette.

pCasper-attB-nos-2×PCP2×mCherry-His2Av-eBFP2 and pHsp83-MCPeGFP were inserted into attP40 [stock 13-20 from the University of Cambridge fly facility: *y w M(eGFP, vas-int, dmRFP)ZH-2A; P{CaryP}attP40*], and pCasper-attB-nos-2×MCP2×eGFP was inserted into attP51C [BDSC: 24482: y^1^ M{vas-int.Dm}ZH-2A w*; M{3×P3-RFP.attP'}ZH-51C]. These were recombined to generate a line expressing both coat proteins and histone marker on the second chromosome: *His2Av-eBFP2, 2×PCP2×mCherry, 2×MCP2×eGFP*.

pUASp-attB-2×MCP2×eGFP-His2Av-eBFP2, pUASp-attB-2×PCP-2×mCherry-His2Av-eBFP2, pUASt-attB-2×MCP2×eGFP and pUASt-attB-2×PCP2×mCherry were inserted into attP86Fb (BDSC: 24749) on the third chromosome. The X chromosome 3×P3-RFP and -GFP markers were selected against after selecting for successful transformants and the landing site 3×P3-RFP was removed using Cre recombinase either before or after injections.

2×MCP2×mNeonGreen was randomly inserted using P-element-mediated transgenesis as described above into the w[*]; P{w[+mC]=His2Av-mRFP1}II.2 line (BDSC:23651) and a single third chromosome insertion stock was found after screening using inverse PCR P-element mapping.

See Table S3 for the stock combinations that were made from these fluorescently tagged coat protein insertions.

### Genomic DNA extractions and PCR screening

Genomic DNA was extracted from 10-15 adult flies. Screening for RMCE events was performed via PCR as described by Venken et al. (2011) using MIL_F and MIL_R primers in combination with the attB_MS2_2 and a primer specific for the loop type. Primers were designed to overlap the pBS-KS-attB1-2 plasmid/loop sequence boundary to avoid the most repetitive sequences of the intervening stem-loop sequences. All primer sequences are listed in Table S1. PCR cycling conditions were as follows: 95°C for 2 min, then 30 cycles of 95°C for 30 s, annealing at 60°C for 30 s, 30 s extension at 72°C using GoTaq polymerase (Promega, M7122). A final 5-min extension of 72°C completed the PCR reactions. As the loops are composed of repetitive sequences, PCR-based methods of screening using primers in these regions can potentially lead to laddering and nonspecific products whereby the newly synthesised PCR products can produce a range of sizes by acting as primers in the subsequent rounds of primer annealing and amplification. We recommend using a PCR cycling protocol with a short annealing time and a low number of cycles, as described here, in the first round of screening.

### Crossing scheme and heat-shock

For second and third chromosome targeting, 30 virgin adult females containing the loop cassette were crossed to 15 males with the MiMIC insertion in the gene of interest in single vials. For X chromosome insertions, 30 virgin adult females containing the MiMIC insertion were crossed to 15 males with the loop cassette in single vials. After allowing flies to lay on the food for 3 days (days 1-3), the flies were transferred onto fresh food. Following removal of adult flies on day 3, the vial containing embryos and larvae aged 1- to 3-days old was immediately heat-shocked in a water bath at 37°C for 30 min. The same vial was then heat-shocked again on day 5 and 7. All crosses were kept at 18°C until heat-shock and the vials with progeny were shifted to 25°C after heat shock until adults emerged. A single 30-min heat shock on day 3 was also successful.

Once adults had emerged, the mosaic eyed *y*^+^ progeny were then crossed to a balancer stock. In the next generation, *yw* males were selected, balanced and screened for direction of the stem-loop insert for autosomal insertions. For insertions on the X chromosome, *yw* males must be selected for and additionally *ry*^+^ should be selected against. The *vasa* phiC31 integrase insertion site is also marked by a 3×P3-RFP, which can be selected against instead of *ry^+^* in the progeny at the final step if preferred.

For X chromosome targeting, the donor cassette flies, which have the heat shock-FLP recombinase and vasa-integrase, were crossed in from the male parent and the female parent provided the MiMIC insertion. This avoids potential viability issues arising from lethal insertions on the X chromosome in the male. Therefore, in the case of X chromosome targeting, recombination can occur between the MiMIC containing *yellow*^+^ locus and the *yellow*^−^ mutation at the endogenous locus in the parental loop donor cassette stock. This may result in *yellow*^−^ progeny emerging at the end of the crossing scheme that do not have the loops inserted into the MiMIC and are instead products of recombination between the two loci. We found that setting up many crosses allowed us to obtain enough progeny to get positive hits from PCR screening even when accounting for recombination between the *roX1* locus and *yellow*. For MiMIC insertions on the X chromosome located far away from the yellow gene, more crosses will need to be set up. In some rare cases, it may be difficult to target the gene of interest if it sits far from the *yellow* locus on the X chromosome, but we anticipate that with enough progeny a successful insertion will occur.

### Embryo and tissue smFISH and immunofluorescence

Embryos were laid on apple juice agar plates supplemented with yeast paste in a 25°C incubator for 2 h then aged for another 2 h. Embryos were then dechorionated in 50% bleach solution (2.5% final concentration of sodium hypochlorite solution diluted in water) and washed thoroughly in ddH_2_O. Embryos were transferred into 3 ml of fixation buffer (1.3×PBS, 67 mM EGTA, pH 8) in a scintillation vial and 1 ml of 37% formaldehyde and 4 ml of heptane was added. Embryos were fixed for 20 min with shaking at 300 rpm as described (Choi et al., 2016; Kosman et al., 2004). After settling, the lower phase was removed and 8 ml methanol was added and vortexed for 1 min. The upper phase was then removed, and embryos were washed in methanol three times before storage at −20°C.

Dissected tissues were fixed in 4% formaldehyde in 1×PBS for 20 min on a rotating wheel. After fixation, three 5-min washes each in 25%, 50% and 75% methanol in PBT (1×PBS with 0.05% Tween20) were performed. Tissues were washed in 100% methanol for 10 min and then transitioned back to 25% methanol in PBT using three 5-min washes. Tissues were processed immediately following fixation for smFISH.

smFISH and immunostaining was performed on embryos as described (Vinter et al., 2021) with the following modifications. Ovary tissues were incubated overnight at 4°C with primary antibodies: rabbit anti-Fibrillarin (Abcam, ab5821, RRID:AB_2105785, 1:1000) and goat anti-GFP antibody (Abcam, ab6673, RRID:AB_305643, 1:500). Secondary antibody incubation was for 2 h using Alexa Fluor antibodies: donkey anti-rabbit 647 (Thermo Fisher Scientific, A-31573, RRID:AB_2536183, 1:500) and donkey anti-goat 488 (Thermo Fisher Scientific, A-11055, RRID:AB_2534102, 1:500). Just before mounting, ovaries were pipetted up and down with a P1000 tip to separate the ovarioles before mounting on slides in Prolong Diamond Antifade Mountant (Thermo Fisher Scientific, P36961). smFISH probe sequences are listed in Table S2.

### Live-imaging microscopy

Embryos were laid on apple juice agar plates supplemented with yeast paste in a 25°C incubator for approximately 1 h and aged if required to just before the desired age. Embryos were collected and dechorionated in 50% bleach solution (2.5% final concentration of sodium hypochlorite solution diluted in water). FluoroDish tissue culture dishes with a cover-glass bottom (World Precision Instruments, FD3510-100) were coated with a thin layer of heptane glue and embryos were mounted onto the heptane glue-coated dishes. A drop of Halocarbon oil (7:1 mix of 700:27 Halocarbon oil; Sigma-Aldrich, H8898 and H8773) was applied over the embryos to keep them from drying out during the imaging period.

For live imaging of wing discs, wandering third instar larvae were dissected in Grace's insect media (Sigma-Aldrich, G8142-500ML) supplemented with 5% fetal bovine serum and 1% Pen/Strep as described (Dye, 2022). Dissected tissues were then transferred into FluoroDish tissue culture dishes in Grace's media for imaging. Ovaries were dissected from 5- to 7-day-old female flies and mounted onto FluoroDish tissue culture dishes as described (Wilcockson and Ashe, 2021).

Images were collected on an Andor Dragonfly200 spinning disc inverted confocal microscope with a Piezo stage using either a 40×/1.30 Super fluor or 100×/1.4 Plan Apo VC objective. Samples were excited using a combination of 405 nm (5-10%), 488 nm (10%) and 561 nm (10-20%) diode lasers and 450 nm DAPI, 525 nm GFP or 600 nm RFP filters, respectively. The laser power and exposure times were set as to not overexpose or bleach the fluorescent signal and differed between genotypes. Each channel was collected sequentially with an iXon EMCCD (1024×1024) camera with a 100-150 ms exposure time per channel. For each movie, a total of 30-50 *z*-stacks at 0.5 µm spacing were collected continuously using the fastest setting yielding a total *z*-size of 15-25 µm at a time resolution of 20-25 s on average.

### Fixed-imaging microscopy

Confocal *z*-stack images of fixed embryos and wing disc tissues were collected using an Andor Dragonfly200 spinning disc inverted confocal microscope as above with 2× or 4× averaging and a combination of 405 nm (5-10%), 488 nm (10%), 561 nm (10-20%) and 637 (15%) diode lasers and 405 nm DAPI, 488 nm GFP, 561 nm RFP or 637 Cy5 filters, respectively, using system-optimised step size. Confocal *z*-stack images of ovaries were collected using a Leica gSTED SP8 inverted confocal microscope using an HC PL APO CS2 100×/1.40 oil objective with a pinhole of 1 airy units, 3.5× zoom, bidirectional scan speed 400 Hz, with an 8 bit image of size 2048×2048 pixel and 4× line averaging at a system-optimised step size. Images were collected by illumination with a white light laser at 70% with the following detection settings: PMT DAPI excitation at 15% 405 nm (collection: 415 to 482 nm); Hybrid Detectors: Quasar 570 excitation at 20% 548 nm (collection: 565 to 631 nm), Quasar 670 excitation at 20% 647 nm (collection: 657 to 735 nm) with 1-6 ns gating.

### Image processing and analysis

Fiji (Schindelin et al., 2012) was used for image processing to crop and make maximum projections of confocal images and time-lapse movies. GraphPad Prism 9 and R studio software were used for plotting and data analysis. Cartoon images of *Drosophila* and vials in figures were made in BioRender.

### Transcription site detection and tracking

Imaris 10.1 (Bitplane) was used to segment nuclei and TSs using the ‘surface’ function for nuclei and the ‘spot’ function for TSs. Spots had an *xy* diameter of 1 µm and *z* diameter of 2 µm. Autoregression tracking of the nuclei used a maximum distance of 5 µm and maximum gap size of 3 time points. Background spots of the same size as the TS spots were added at every third time point. Nuclei and spot statistics were run through the sass algorithm (Hoppe and Ashe, 2021b), which assigns spots to nuclei over time with background subtraction to give background corrected fluorescent intensity of each TS over time assigned to its nearest nucleus.

For movies of follicle cells and wing discs, the TSs were detected as ‘spots’ as described above but with tracking using autoregressive motion and a maximum distance of 1.5 µm and gap size of 3 time points. Background intensity was calculated by doubling the volume of each of the spots over time and subtracting the spot intensity sum from the double spot volume intensity sum. This background intensity was then subtracted from each spot intensity sum for each transcription site ‘spot’ to give a background-subtracted spot sum intensity at each time point.

## Supplementary Material

10.1242/develop.204294_sup1 Supplementary information

Table S1. Table of PCR primers used in this study.

Table S2. Table of smFISH probes used in this study.
