# Supplementary material for: A simple MiMIC-based approach for tagging endogenous genes to visualise live transcription in Drosophila
Source: Development. 2024 Dec 16;151(24):dev204294. doi: 10.1242/dev.204294 (PMC11701513; doi:10.1242/dev.204294)
Supplement: Supplementary information [file develop-151-204294-s1.pdf]

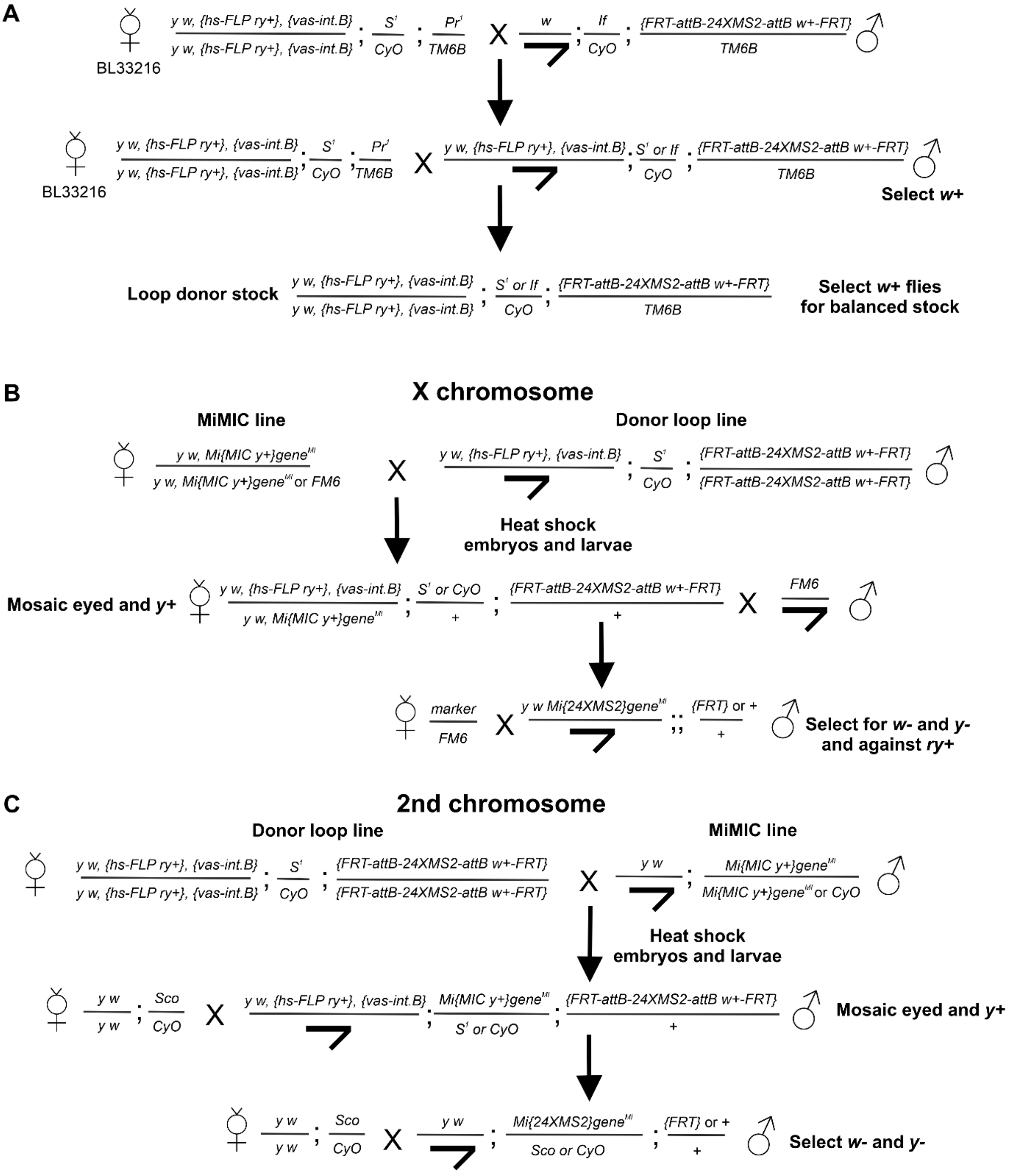


**Fig. S1. Crossing scheme for inserting loop sequences into genes on the X and second chromosome.**

(A) Crossing scheme for crossing donor loop lines into the background of *hsFLP, vasa-integrase* flies, BL33216. (B, C) Crossing scheme for insertion of 24xMS2 loops into a MiMIC on the X (B) and second (C) chromosome.

Development • Supplementary information

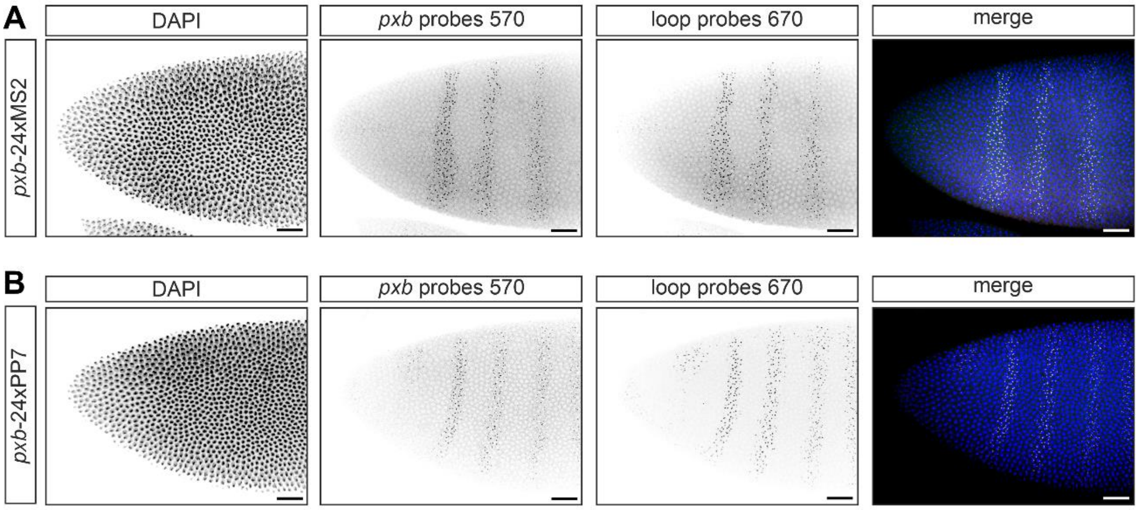


**Fig. S2. *pxb* transcription in embryos endogenously tagged with 24xMS2 or 24xPP7 loops.**

(A) Confocal images of *pxb-24xMS2* embryos showing the anterior stripes of the *pxb* expression domain visualised by smFISH staining with *pxb* probes (570 channel, green) and MS2 loop probes (670 channel, magenta). Nuclei are labelled with DAPI (blue). (B) As in (A) except that *pxb-24xPP7* embryos were stained and PP7 smFISH probes were used. Scale bar is 50μm.

Development • Supplementary information

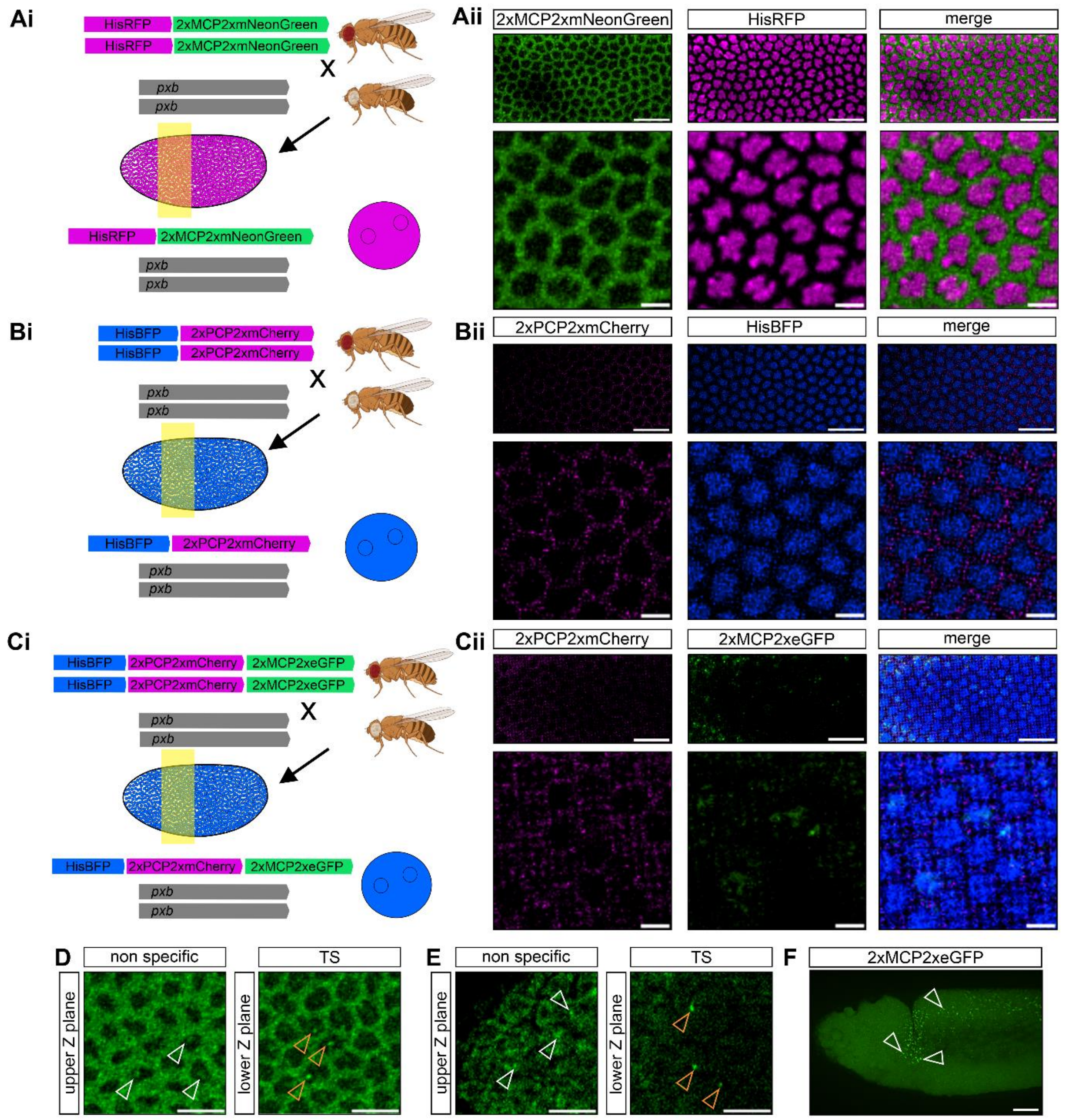


**Fig. S3. Control embryos showing fluorescent coat protein signals in the absence of loops.** (Ai) *HisRFP; 2xMCP2xmNeonGreen* females are crossed to control male flies with no loops inserted in the *pxb* gene. Embryos have no *pxb* alleles visible within the HisRFP nucleus, the imaging region is shown in yellow corresponding to the same region in Figure 3. (Aii) Top: Still images from a timelapse movie of nc14 embryos with maternally loaded HisRFP and 2xMCP2xmNeonGreen. Scale bar is 20μm. Bottom: Higher magnification image from nuclei within the region. Nuclei are marked in magenta, 2xMCP2xmNeonGreen is in green. Scale bar is 5μm. (Bi) As in (Ai) but from *HiseBFP2, 2xPCP2xmCherry* females crossed to control male flies. (Bii) As in (Aii) but from an embryo maternally loaded with HiseBFP2 and 2xPCP2xmCherry marking the nuclei in blue and the 2xPCP2xmCherry signal in magenta. (Ci) As in (Ai) but from *HiseBFP2, 2xPCP2xmCherry, 2xMCP2xeGFP* females crossed to

Development • Supplementary information

control male flies. (Cii) As in (Aii) but embryos have maternally loaded HiseBFP2, 2xPCP2xmCherry and 2xMCP2xeGFP fluorescent coat proteins. 2xPCP2xmCherry is in magenta, 2xMCP2xeGFP is in green, nuclei are blue in the merge. (D) Different Z slices from the embryo in Fig 3Aii showing non-specific 2xMCP2xmNeonGreen signals (white arrowheads) in the apical region (upper Z plane) compared to a lower Z plane where the *pxb* TSs are located (orange arrowheads). Scale bar is 10µm. (E) As in (D) but for 2xMCP2xeGFP embryos from Fig 3Cii. (F) Still from a live movie showing aggregation of 2xMCP2xeGFP in a gastrulating embryo laid from homozygous *HiseBFP2, nos-2xPCP2xmCherry, nos-2xMCP2xeGFP* females. Scale bar is 30µm. See also Movies 4-6. Fly cartoons were created in BioRender. Ashe, H. (2024) https://BioRender.com/u96a435.

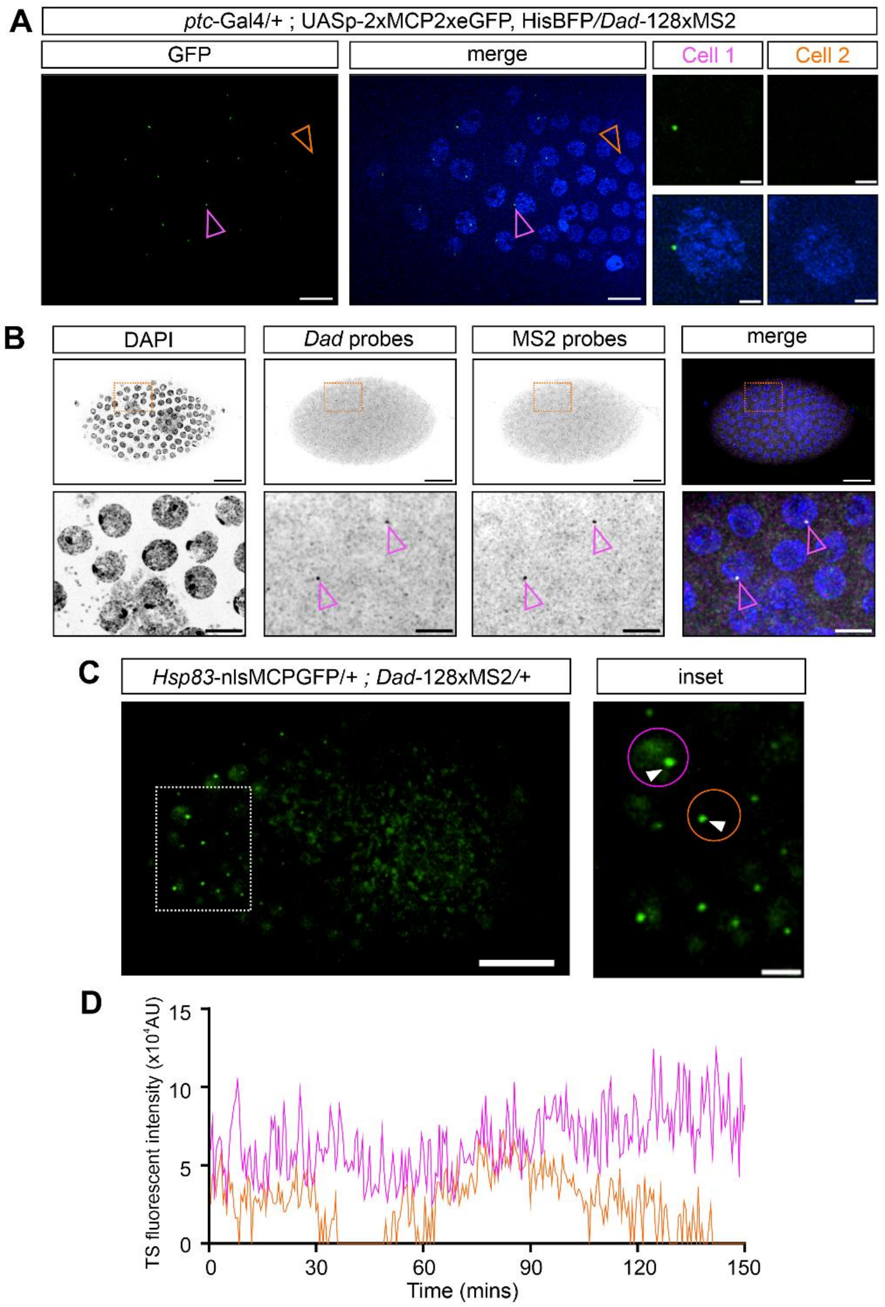


**Fig. S4. Transcription of *Dad-128xMS2* in ovarian follicle cells.**

(A) Still from a live imaging movie of a stage 8 egg chamber from a *ptc-Gal4/+ ; UASp-2xMCP2xeGFP, HiseBFP2/Dad-128xMS2* female, anterior is to the left. *Dad-128xMS2* is transcribed in the anterior follicle cells (magenta arrow). Posterior cells do not have *Dad-128xMS2* TSs (orange arrow). Higher magnification images of two highlighted cells are shown on the right. (B) (Top) Confocal images of stage 8 egg chambers showing *Dad-128xMS2* transcription in follicle cells. Nuclei are stained with DAPI and show smFISH detection of *Dad*

Development • Supplementary information

and MS2 loops. A highlighted region of interest (orange box) is shown below. (Bottom) Higher magnification images of cells from the boxed area, TSs are marked with arrowheads. Scale bar is 20µm (top) and 5µm (bottom). (C) Still from a live imaging movie of a stage 8 egg chamber from a *Hsp83-nlsMCPeGFP/+ ; Dad-128xMS2/+* female, anterior is to the left. *Dad* is transcribed in the anterior follicle cells with a region of expressing cells marked by the white box. Inset: A higher magnification image of the follicle cells highlighting two cells (purple and orange circles) with TSs (arrowheads). (D) Fluorescent intensity traces from the two cells highlighted in the inset.

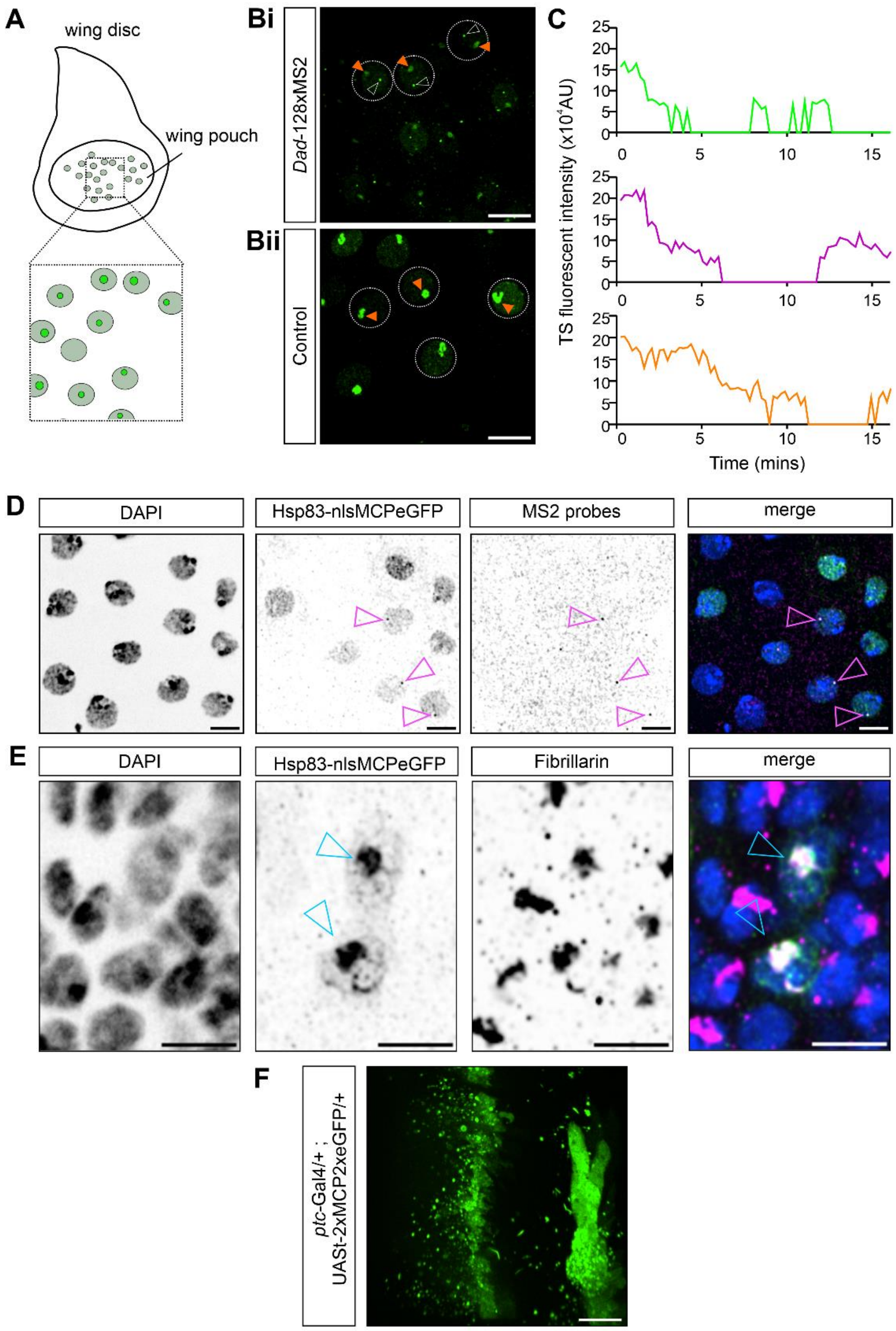
A
wing disc
wing pouch
Bi
Dad-128xMS2
Bii
Control
C
TS fluorescent intensity (x10⁴AU)
Time (mins)
D
DAPI
Hsp83-nlsMCPeGFP
MS2 probes
merge
E
DAPI
Hsp83-nlsMCPeGFP
Fibrillarin
merge
F
ptc-Gal4/+ ;
UASt-2xMCP2xeGFP/+

**Fig. S5. Visualising nascent transcription in the wing disc**.

(A) Schematic showing the third instar larval wing disc highlighting the peripodial cells of the wing pouch that are imaged live. (B) Still from a live imaging movie of cells from *Hsp83-nlsMCPeGFP/+ ; Dad-128xMS2/+* (Bi) and control (Bii) larvae lacking the 128xMS2 cassette. The outlined cells show active TSs (white arrowheads) and non-specific accumulation of MCPeGFP in the nucleoli (orange arrowheads). Scale bar is 5µm. (C) TS traces from the outlined cells in (Bi) showing transcriptional bursting. (D) Confocal images of *Dad-128xMS2* transcription in wing disc cells from *Hsp83-nlsMCPeGFP ; Dad-128xMS2* 3$^{rd}$ instar larva. Nuclei are stained with DAPI and show MCPeGFP marked *Dad* TSs and smFISH detection of MS2 loops. TSs are labelled with arrowheads. Scale bar is 5µm. (E) Hsp83-nlsMCPeGFP expressing cells of the wing disc without any MS2 loop sequences. MCPeGFP accumulation colocalises with the nucleolus marked by anti-Fibrillarin immunofluorescence. Scale bar is 5µm. (F) Still from a live imaging movie of a wing disc from a *ptc-Gal4/+; UASt- 2xMCP2xeGFP/+* 3$^{rd}$ instar larvae showing aggregation of 2xMCP2xeGFP within the *ptc* domain. Scale bar is 20µm.

Development • Supplementary information

## Table S1. Table of PCR primers used in this study.

Available for download at
https://journals.biologists.com/dev/article-lookup/doi/10.1242/dev.204294#supplementary-data

## Table S2. Table of smFISH probes used in this study.

Available for download at
https://journals.biologists.com/dev/article-lookup/doi/10.1242/dev.204294#supplementary-data

## Table S3. Fly stocks used and made in this study

| Genotype | Source | Identifier |
|---|---|---|
| P{ry[+t7.2]=hsFLP}12, $y^1$ $w^*$ M{vas-int.B}ZH-2A; $Pri^1$/TM3, $Sb^1$ | Bloomington Drosophila Stock Center | BSDC:33216 |
| $y^1$ $w^*$; $nub^2$ $b^1$ $sna^{Sco}$ $pr^1$ $cn^1$/CyO | Bloomington Drosophila Stock Center | BSDC:3628 |
| $y^1$ $w^*$; Mi{y[+mDint2]=MIC}pxb[MI04897] | Bloomington Drosophila Stock Center | BSDC:37864 |
| $y^1$ $w^*$; Mi{y[+mDint2]=MIC}Ance[MI05748]/SM6a | Bloomington Drosophila Stock Center | BSDC:42109 |
| $y^1$ $w^*$ Mi{y[+mDint2]=MIC}lncRNA:roX1[MI01457] | Bloomington Drosophila Stock Center | BSDC:44848 |
| $y^1$ w*; Mi{y[+mDint2]=MIC}Dad[MI04922] | Bloomington Drosophila Stock Center | BSDC:37913 |
| $y^1$ $w^{67c23}$; $sna^{Sco}$/CyO, P{Crew}DH1 | Bloomington Drosophila Stock Center | BSDC:1092 |
| $w^*$; If/CyO; MKRS/TM6B | University of Manchester fly facility | |
| $y^1$ $w^{67c23}$ | Bloomington Drosophila Stock Center | BSDC:6599 |
| *w*$^{1118}$ | Bloomington Drosophila Stock Center | BSDC:5905 |
| $y^1$ $w^*$;; CxD/TM6C | This study | |
| w*; P{w[+mC]=His2Av-mRFP1}II.2 | Bloomington Drosophila Stock Center | BSDC:23651 |
| $y^1$ w*; P{w[+mC]=Hsp83-MCPeGFP}5A (referred to here as Hsp83-nlsMCPeGFP) | Bloomington Drosophila Stock Center | BSDC:7279 |
| $y^1$ M{vas-int.Dm}ZH-2A w*; M{3xP3-RFP.attP'}ZH-51C | Bloomington Drosophila Stock Center | BSDC:24482 |
| $y^1$ M{RFP[3xP3.PB] GFP[E.3xP3]=vas-int.Dm}ZH-2A w*; M{3xP3-RFP.attP}ZH-86Fb | Bloomington Drosophila Stock Center | BSDC:24749 |
| y w M(eGFP, vas-int, dmRFP)ZH-2A; P{CaryP}attP40 | University of Cambridge fly facility | 13-20 |
| $y^1$ w*; his2Av-eBFP2, nos-2xPCP2xmCherry | This study | |
| P{w[+mC]=His2Av-mRFP1}II.2; nos-2xMCP2xmNeonGreen | This study | |
| $y^1$ w*; his2Av-eBFP2, nos-2xPCP2xmCherry, nos-2xMCP2xeGFP | This study | |
| w*; his2Av-eBFP2/CyO; nos-2xMCP2xmCherry/TM6b | This study | |

Development • Supplementary information

| | | |
|---|---|---|
| P{ry[+t7.2]=hsFLP}12, y[1] w[*] M{vas-int.B}ZH-2A; 24xPP7/CyO; Pri[1]/TM6B, Tb[1] | This study | |
| P{ry[+t7.2]=hsFLP}12, y[1] w[*] M{vas-int.B}ZH-2A; If/CyO; 24xPP7/TM6B, Tb[1] | This study | |
| P{ry[+t7.2]=hsFLP}12, y[1] w[*] M{vas-int.B}ZH-2A; S[1]/CyO; 24xMS2V5/TM6B, Tb[1] | This study | |
| P{ry[+t7.2]=hsFLP}12, y[1] w[*] M{vas-int.B}ZH-2A; 24xMS2V6/CyO; Pri[1]/TM6B, Tb[1] | This study | |
| P{ry[+t7.2]=hsFLP}12, y[1] w[*] M{vas-int.B}ZH-2A; 128xMS2/CyO; Pri[1]/TM6B, Tb[1] | This study | |
| P{ry[+t7.2]=hsFLP}12, y[1] w[*] M{vas-int.B}ZH-2A; S[1]/CyO; 128xMS2/TM6B, Tb[1] | This study | |
| P{ry[+t7.2]=hsFLP}12, y[1] w[*] M{vas-int.B}ZH-2A; 24xMS2-SL/CyO; Pri[1]/TM6B, Tb[1] | This study | |
| $y^{1}$ w*;;UASp-2xMCP2xeGFP his2Av-eBFP2 | This study | |
| $y^{1}$ w*;;UASt-2xMCP2xeGFP | This study | |
| $y^{1}$ w*;;UASp-2xPCP2xmCherry his2Av-eBFP2 | This study | |
| $y^{1}$ w*;;UASt-2xPCP2xmCherry | This study | |
| Hsp83-MCPeGFP | This study | |
| w*; P{w[+mW.hs]=GawB}ptc[559.1] | Bloomington Drosophila Stock Center | BSDC:2017 |

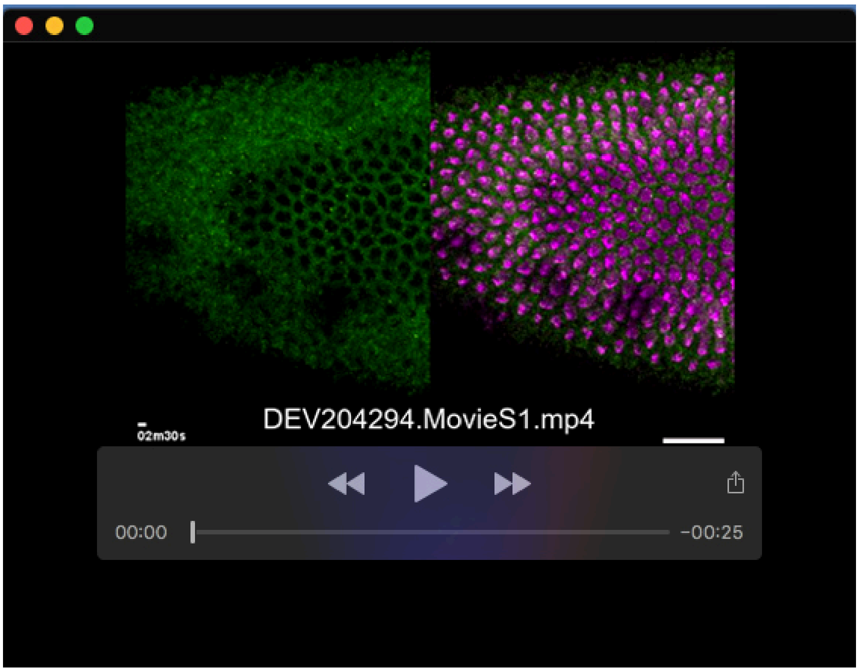


**Movie 1. Live imaging of *pxb-24xMS2* in a nuclear cycle 14 *HisRFP/+; nos-2xMCP2xmNeonGreen/pxb-24xMS2* embryo.** Scale bar is 20µm.

Development • Supplementary information

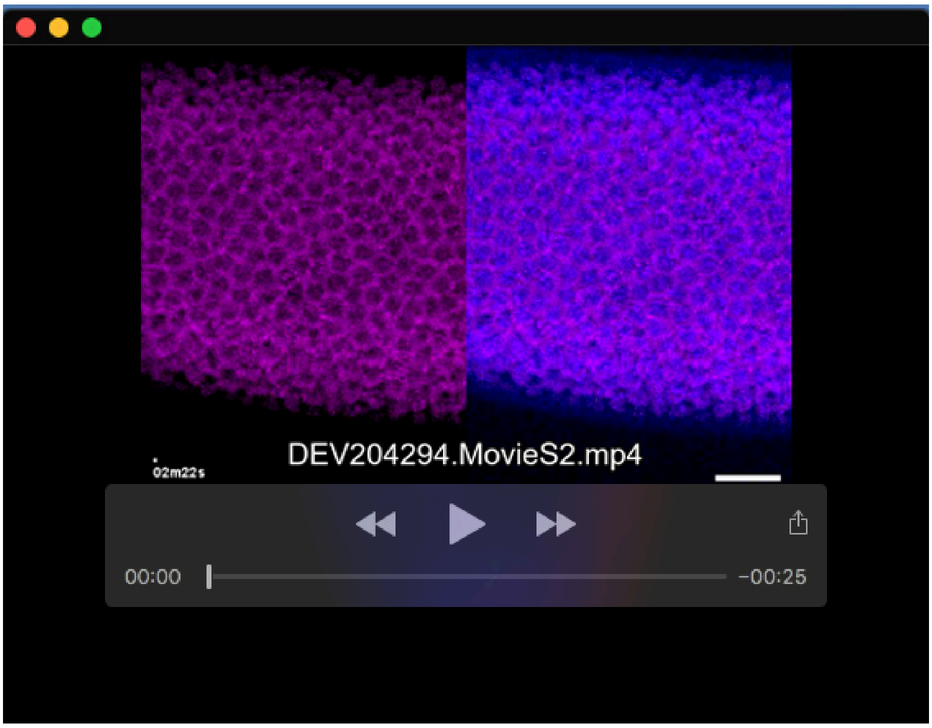


**Movie 2. Live imaging of *pxb*-24xPP7 in a nuclear cycle 14 *HiseBFP2, nos-2xPCP2xmCherry/+; pxb-24xPP7/+* embryo.** Scale bar is 20μm.

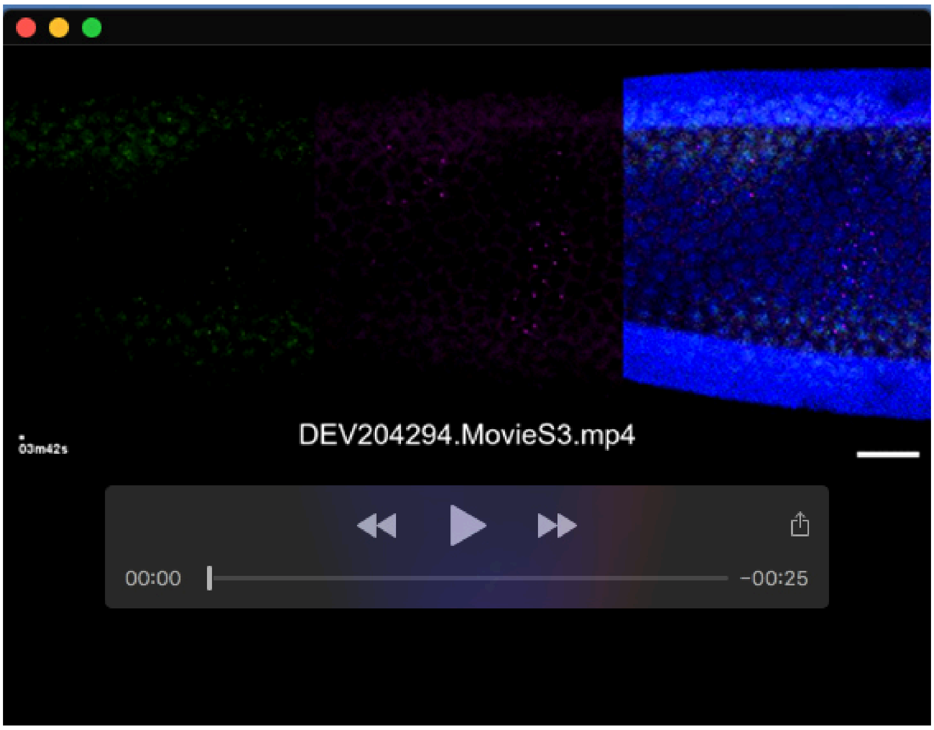


**Movie 3. Live imaging of *pxb*-24xMS2 and *pxb*-24xPP7 in a nuclear cycle 14 *HiseBFP2, nos-2xPCP2xmCherry, nos-2xMCP2xGFP/+; pxb-24xMS2/pxb-24xPP7* embryo.** Scale bar is 20μm.

Development • Supplementary information

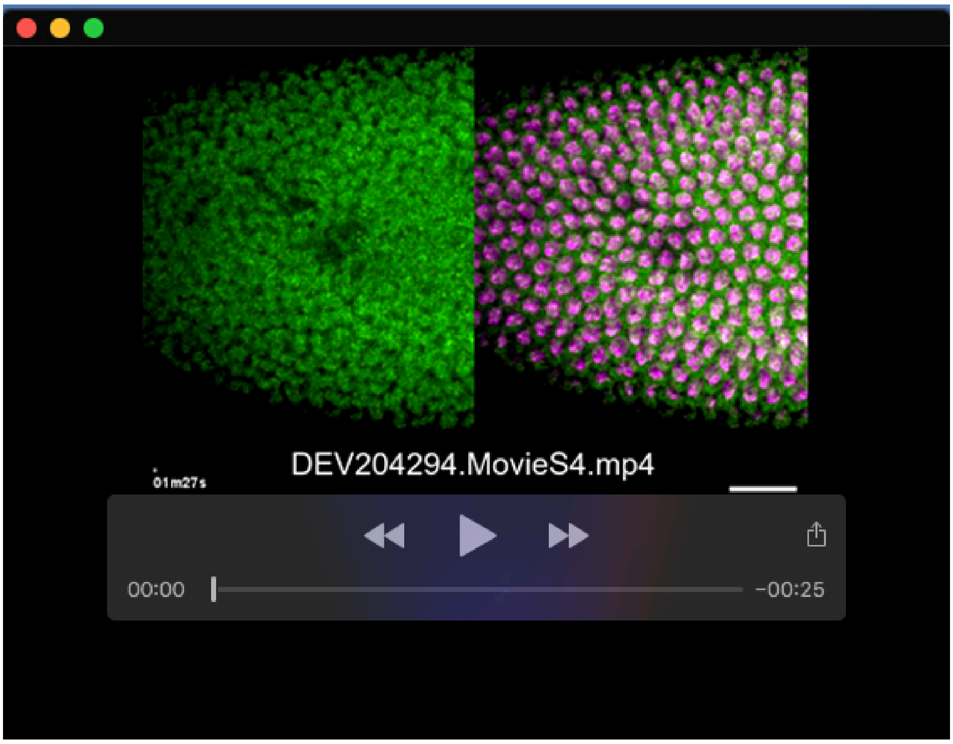


**Movie 4. Live imaging of a control *HisRFP/+; nos-2xMCP2xmNeonGreen/+* embryo.** Scale bar is 20μm.

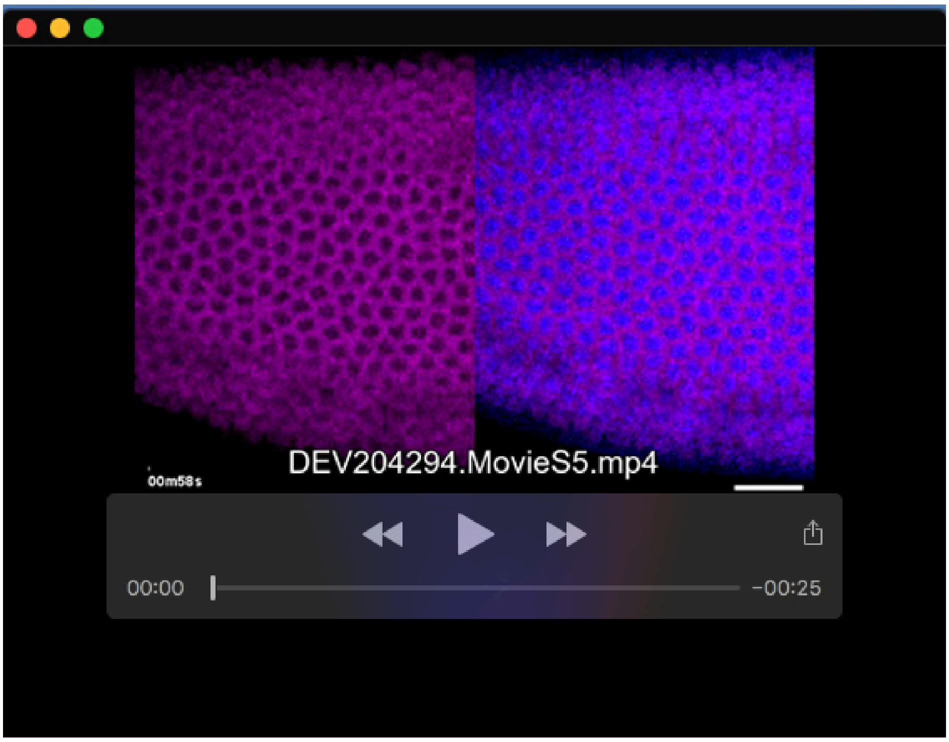


**Movie 5. Live imaging of a control *HiseBFP2, nos-2xPCP2xmCherry/+* embryo.** Scale bar is 20μm.

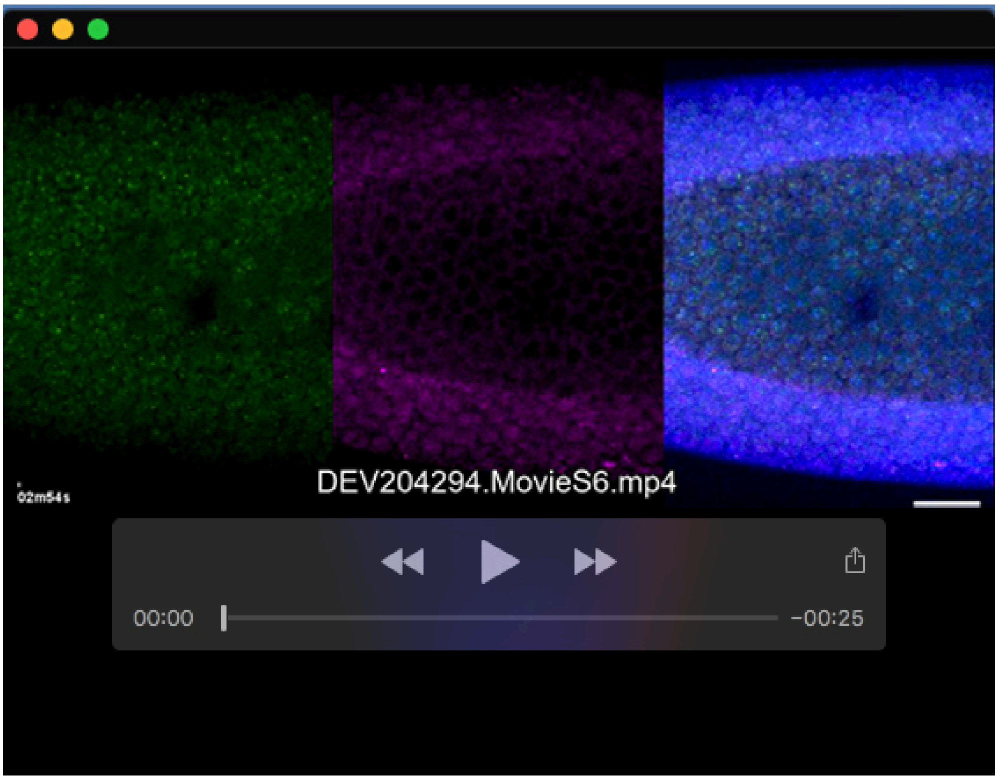


**Movie 6. Live imaging of a control *HiseBFP2, nos-2xPCP2xmCherry, nos-2xMCP2xeGFP/+* embryo.** Scale bar is 20μm.

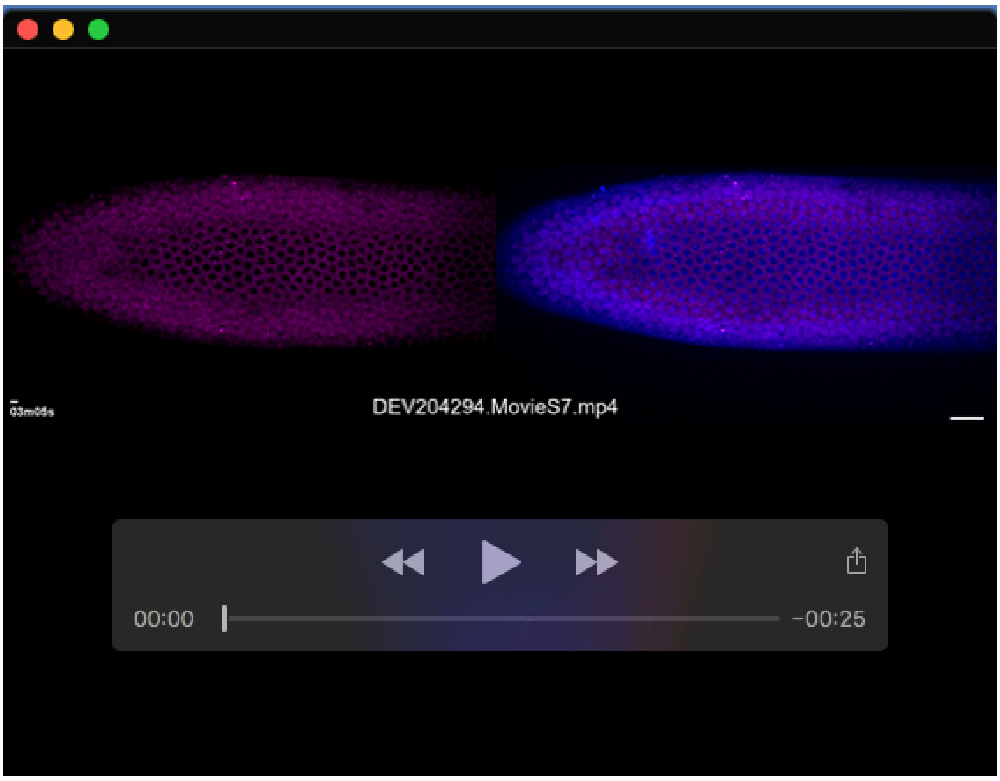


**Movie 7. Live imaging of *Race-24xPP7* in a nuclear cycle 14 *HiseBFP2, nos-2xPCP2xmCherry/Race-24xPP7* embryo.** Scale bar is 20μm.

Development • Supplementary information

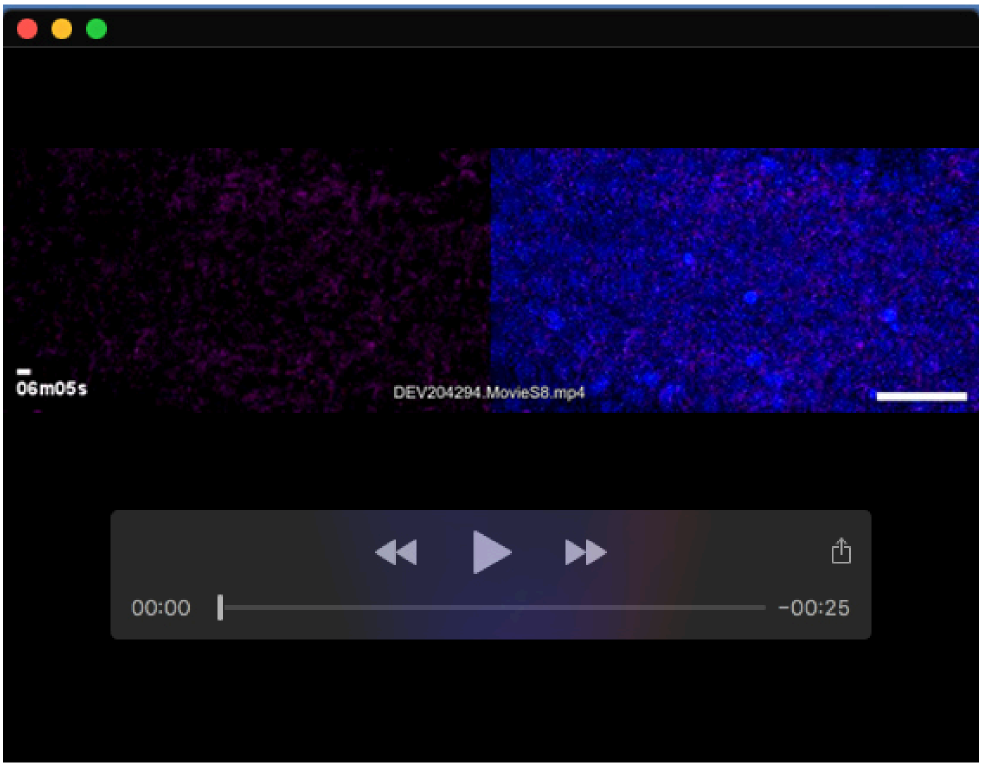


**Movie 8. Live imaging of *roX1-24xPP7* in a nuclear cycle 14 *roX1-24xPP7/Y; HiseBFP2, nos-2xPCP2xmCherry/+* embryo.** Scale bar is 20μm.

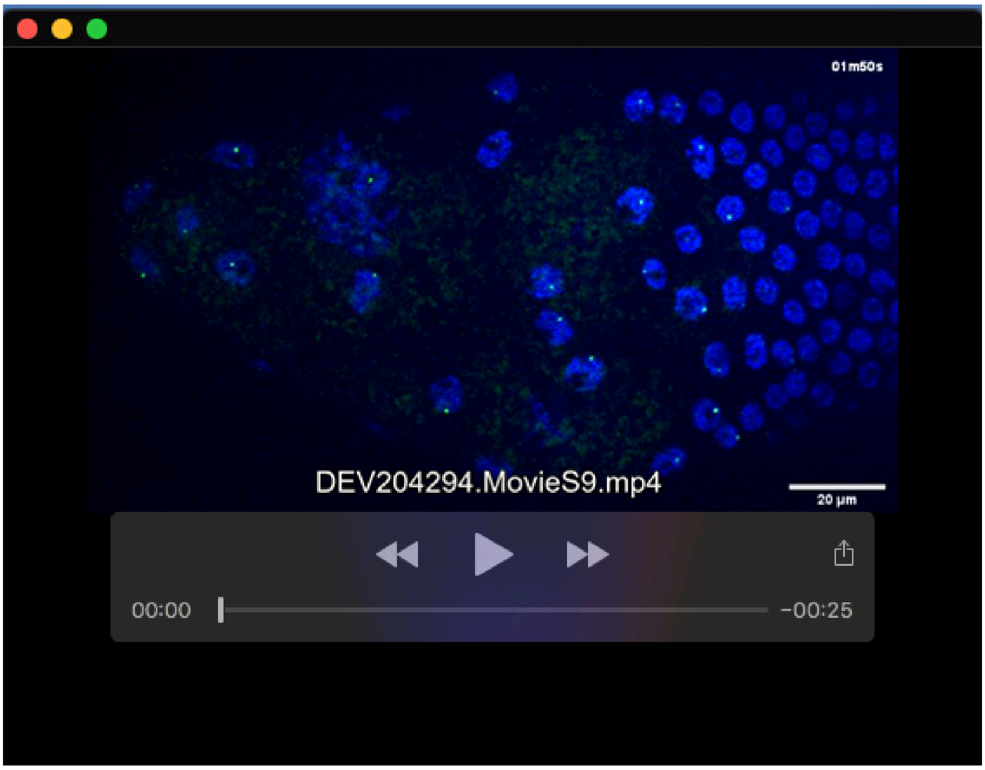


**Movie 9. Live imaging of *Dad-128xMS2* in the follicle cells of stage 8 egg chambers from female *ptc-Gal4/+; UASp-2xMCP2xeGFP, HiseBFP2/Dad-128xMS2* flies.**

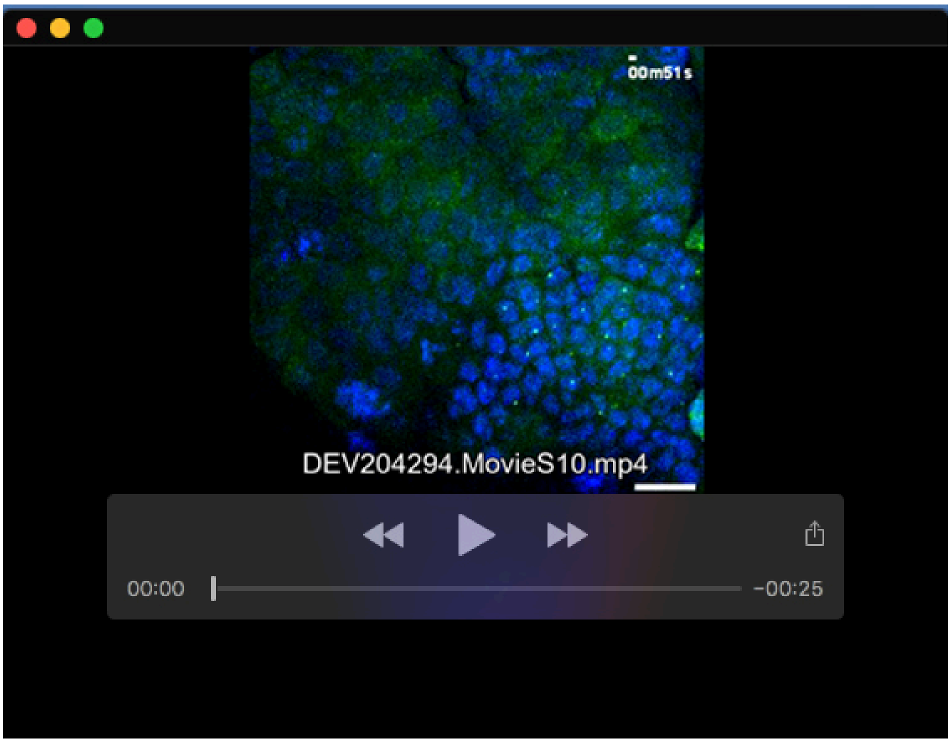


**Movie 10. Live imaging of *Dad-128xMS2* in the larval brain from *ptc-Gal4/+; UASp-2xMCP2xeGFP, HiseBFP2/Dad-128xMS2*.** Scale bar is 15μm.

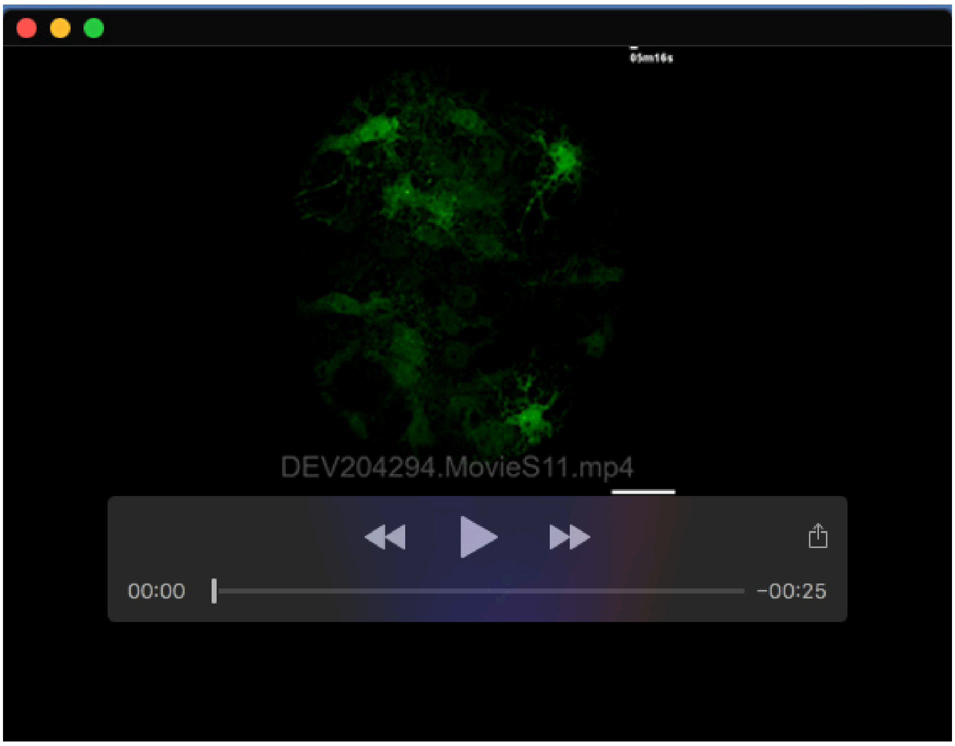


**Movie 11. Live imaging of *Dad-128xMS2* in the larval brain using Hsp83-MCPeGFP from *Hsp83-MCPeGFP/+; Dad-128xMS2/+* larvae.** Scale bar is 20μm.

Development • Supplementary information

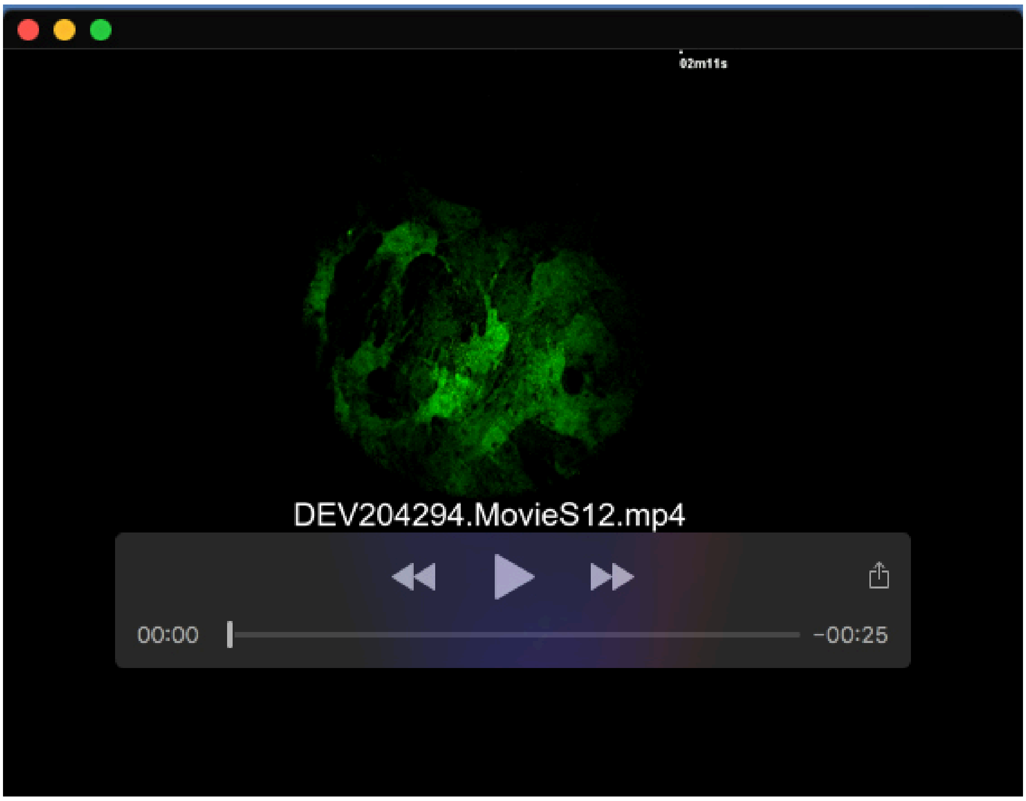


**Movie 12. Live imaging of brains from control *Hsp83-MCPeGFP/+* larvae.** Scale bar is 20µm.
